# Nr2f-dependent allocation of ventricular cardiomyocyte and pharyngeal muscle progenitors

**DOI:** 10.1371/journal.pgen.1007962

**Published:** 2019-02-05

**Authors:** Tracy E. Dohn, Padmapriyadarshini Ravisankar, Fouley T. Tirera, Kendall E. Martin, Jacob T. Gafranek, Tiffany B. Duong, Terri L. VanDyke, Melissa Touvron, Lindsey A. Barske, J. Gage Crump, Joshua S. Waxman

**Affiliations:** 1 Division of Molecular Cardiovascular Biology, Cincinnati Children’s Hospital Medical Center, Cincinnati, OH, United States of America; 2 Molecular and Developmental Biology Graduate Program, University of Cincinnati College of Medicine, Cincinnati, OH, United States of America; 3 Master’s Program in Genetics, Department of Life Sciences, Université Paris Diderot, Paris, France; 4 Molecular Genetics and Human Genetics Graduate Program, University of Cincinnati College of Medicine, Cincinnati, OH, United States of America; 5 Molecular and Developmental Biology Master’s Program, University of Cincinnati College of Medicine, Cincinnati, OH, United States of America; 6 Eli and Edythe Broad Center for Regenerative Medicine and Stem Cell Research, University of Southern California, Los Angeles, CA, United States of America; 7 Department of Pediatrics, University of Cincinnati College of Medicine, Cincinnati, OH, United States of America; University of Pennsylvania School of Medicine, UNITED STATES

## Abstract

Multiple syndromes share congenital heart and craniofacial muscle defects, indicating there is an intimate relationship between the adjacent cardiac and pharyngeal muscle (PM) progenitor fields. However, mechanisms that direct antagonistic lineage decisions of the cardiac and PM progenitors within the anterior mesoderm of vertebrates are not understood. Here, we identify that retinoic acid (RA) signaling directly promotes the expression of the transcription factor Nr2f1a within the anterior lateral plate mesoderm. Using zebrafish *nr2f1a* and *nr2f2* mutants, we find that Nr2f1a and Nr2f2 have redundant requirements restricting ventricular cardiomyocyte (CM) number and promoting development of the posterior PMs. Cre-mediated genetic lineage tracing in *nr2f1a; nr2f2* double mutants reveals that *tcf21*^*+*^ progenitor cells, which can give rise to ventricular CMs and PM, more frequently become ventricular CMs potentially at the expense of posterior PMs in *nr2f1a; nr2f2* mutants. Our studies reveal insights into the molecular etiology that may underlie developmental syndromes that share heart, neck and facial defects as well as the phenotypic variability of congenital heart defects associated with NR2F mutations in humans.

## Author summary

Many developmental syndromes include both congenital heart and craniofacial defects, necessitating a better understanding of the mechanisms underlying the correlation of these defects. During early vertebrate development, cardiac and pharyngeal muscle cells originate from adjacent, partially overlapping progenitor fields within the anterior mesoderm. However, signals that allocate the cells from the adjacent cardiac and pharyngeal muscle progenitor fields are not understood. Mutations in the gene *NR2F2* are associated with variable types of congenital heart defects in humans. Our recent work demonstrates that zebrafish Nr2f1a is the functional equivalent to Nr2f2 in mammals and promotes atrial development. Here, we identify that zebrafish *nr2f1a* and *nr2f2* have redundant requirements at earlier stages of development than *nr2f1a* alone to restrict the number of ventricular CMs in the heart and promote posterior pharyngeal muscle development. Therefore, we have identified an antagonistic mechanism that is necessary to generate the proper number of cardiac and pharyngeal muscle progenitors in vertebrates. These studies provide evidence to help explain the variability of congenital heart defects from NR2F2 mutations in humans and a novel molecular framework for understanding developmental syndromes with heart and craniofacial defects.

## Introduction

During organogenesis, the initial specification of organ fields generates overlapping populations of progenitor cells that harbor the potential to contribute to multiple organs [1, 2]. In vertebrates, the anterior lateral plate mesoderm (ALPM), which generates the cardiac progenitor field, develops adjacent to the cranial paraxial mesoderm, which generates the pharyngeal muscle (PM) progenitor field, the source of facial and neck muscles [3–5]. In mice, detailed retrospective clonal lineage-tracing has revealed there are rare bi-potent cardio-PM progenitors, which potentially lie at the interface of these progenitor fields and give rise to the heart, pharyngeal, and neck muscles [6–8]. Specifically, craniofacial muscles of the 1^st^ and 2^nd^ pharyngeal arches share progenitors with the right ventricle and outflow tract, respectively [6, 7], which are derivatives of the later differentiating second heart field (SHF) [9, 10]. However, muscles of the neck share progenitors from a distinct later-differentiating SHF population that contributes to the pulmonary arterial pole and atria [8]. Thus, these studies have emphasized the integration of developmental potential that generates multiple cardiac and PM progenitor populations during vertebrate development.

Given the proximity of the cardiac and PM progenitor fields within the anterior mesoderm of vertebrates, there is significant overlap in the expression of conserved regulators of these lineages. The transcription factors Tbx1 and Tcf21, in particular, share expression in cardiac and PM progenitors and are required to promote their development [11–14]. In humans, heterozygosity of *TBX1* underlies DiGeorge Syndrome, which is characterized by congenital outflow tract and craniofacial defects [15]. Furthermore, studies using knockout (KO) mice have demonstrated that Tbx1 is at the top of a complex genetic hierarchy that directs the development of the outflow tract and all PMs [11, 12]. Within this genetic hierarchy, Tcf21 appears to act downstream of Tbx1. Compared to *Tbx1*, loss of *Tcf21* in mice results in less severe outflow tract and PM defects [12], which is likely due to redundancy with *Musculin/MyoR* [16]. As in mammals, zebrafish *tbx1* mutants have outflow tract and craniofacial defects [14, 17, 18]. Furthermore, in zebrafish, *tcf21*^*+*^ progenitors contribute to both ventricular cardiomyocytes (CMs) and PMs [5]. However, in contrast to mice, *tcf21* in zebrafish is required for the development of almost all PMs [5]. Thus, a conserved network of core transcription factors promotes the development of both cardiac outflow tract and PMs in vertebrates.

There is evidence that the origin of bi-potent SHF cardiac and PM progenitors is conserved in chordates [19]. Work in the tunicate *Ciona* has shed some light on transcriptional signals that drive cardiac and PM fate decisions within distinct precursors of the SHF [20]. Despite the conservation of core factors, including Tbx1 and Nkx homologs, there is currently limited understanding of signals that allocate the cardiac and PM lineages through driving differential fate decisions of progenitors from these adjacent organ fields in vertebrates. Retinoic acid (RA) signaling is currently the only known signaling pathway that overtly restricts cardiac specification and promotes craniofacial development in vertebrates [21–26]. However, the mechanisms by which RA signaling may coordinate cardiomyocyte (CM) and PM fate decisions from these progenitor fields within the anterior mesoderm are not understood.

NR2F proteins (formerly called COUP-TFs) are highly conserved orphan nuclear receptor transcription factors whose expression is RA-responsive in many tissues of all vertebrates [27–30]. In mammals, the expression of two NR2F genes, *NR2F1* and *NR2F2*, overlaps during early embryonic development as well as later in atrial CMs of the heart [29–32]. Despite some overlap in limited cell types, expression of these two genes in mice largely diverges after early stages of embryogenesis, with *Nr2f1* and *Nr2f2* becoming predominantly expressed in neural and mesendodermal tissues, respectively [27, 29]. Analysis of individual KO mice has revealed requirements in organs that are consistent with their tissue-specific expression patterns [33–36]. With respect to the heart, global *Nr2f2* knockout (KO) mice have morphologically smaller atria and sinus venosus [35]. Conditional cardiac-specific *Nr2f2* KO mice studies using a *Myh6*:*Cre* suggest a later role for Nr2f2 in maintaining atrial CM identity [36]. While zebrafish *nr2f2* mutants are not early embryonic lethal and do not have overt cardiovascular defects through at least two weeks of development [37, 38], our recent analysis of zebrafish *nr2f1a* mutants indicates that it is the functional homolog of Nr2f2 in mammals with respect to early heart development [39]. Specifically, zebrafish *nr2f1a* mutants have smaller atria due to a requirement within atrial CMs to concomitantly promote atrial differentiation and limit the size of the atrioventricular canal (AVC) [39]. *NR2F1* and *NR2F2* are redundantly required for atrial differentiation in human iPSC-derived atrial cells [32], although NR2F2 seems to have a primary role. Consistent with conserved requirements in vertebrate atrial development, lesions affecting *NR2F2* have been associated with variable types of human congenital heart defects (CHDs), in particular atrial septal defects (ASDs) and atrioventricular septal defects (AVSDs), but surprisingly also left ventricular outflow tract obstruction (LVOTO) [40, 41]. Therefore, while analysis of vertebrate *Nr2f* mutant models has provided insight into the molecular etiology of CHDs affecting the atria and AVC, the mechanisms underlying the observed phenotypic variability of CHDs, in particular the origins of ventricular malformations, in humans with *NR2F2* mutations are not understood.

Here, we identify that RA signaling directly regulates *nr2f1a* expression within the ALPM of zebrafish embryos and that retinoic acid receptors (RARs) can bind an absolutely conserved, yet unconventionally localized, response element. Using zebrafish mutants for both *nr2f1a* and *nr2f2*, we find redundant functions at earlier developmental stages in restricting ventricular CM and promoting PM specification, independent of the later requirement for *nr2f1a* in promoting atrial differentiation. Cre-mediated genetic lineage tracing shows that *tcf21*^*+*^ progenitors more frequently become ventricular CMs and less frequently contribute to skeletal muscle within the posterior PM in *nr2f1a; nr2f2* mutant embryos. Our results support a novel antagonistic mechanism that controls allocation of ventricular CM and PM progenitors within the anterior mesoderm of vertebrates and may help explain the correlation of craniofacial and heart defects as well as the variability found in CHDs associated with *NR2F2* mutations in humans.

## Results

### RA receptors bind a conserved RA response element in the *nr2f1a* promoter

**Fig 1:**
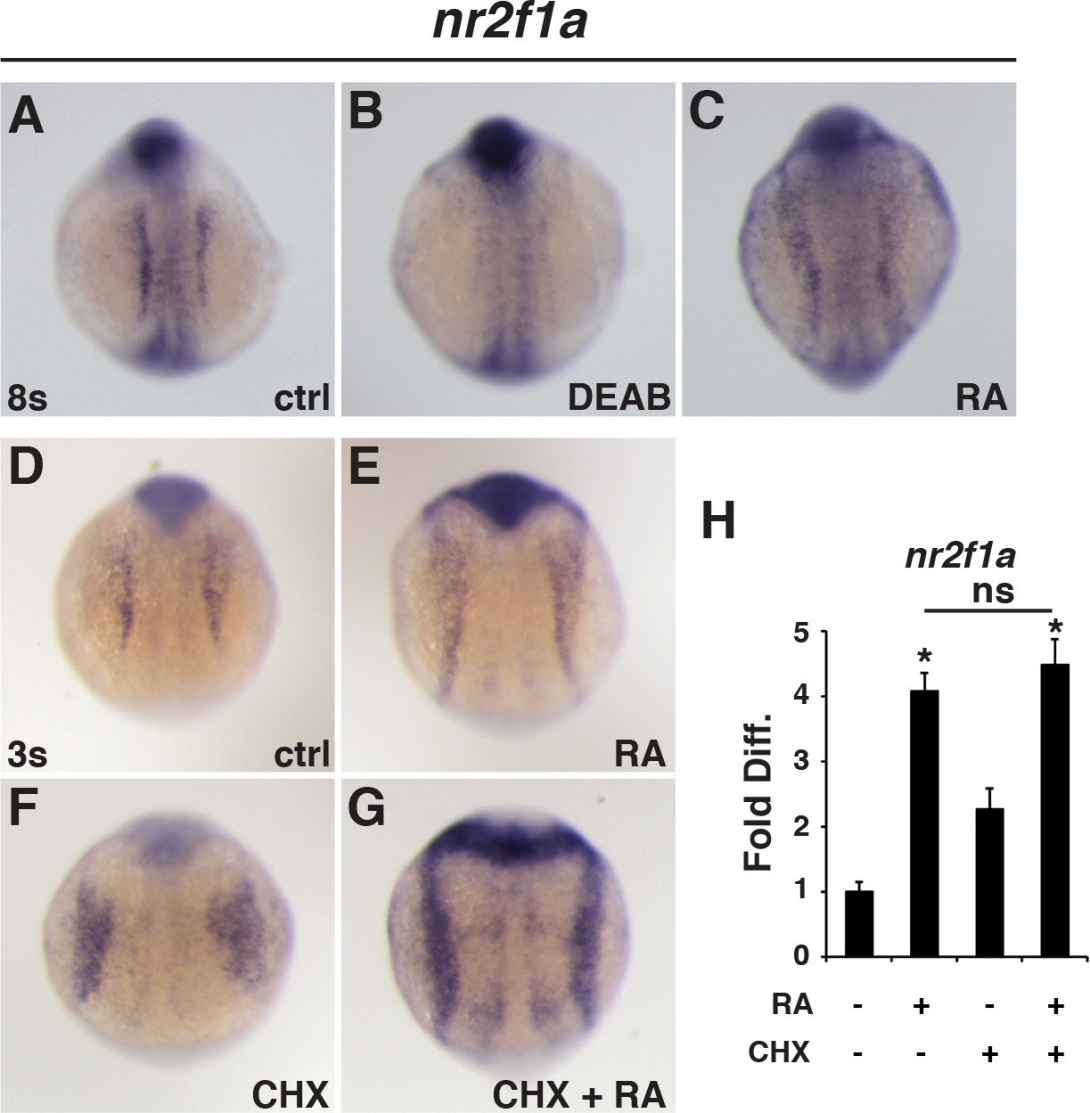
RA signaling directly regulates *nr2f1a* expression. (A-C) *In situ* hybridization (ISH) for *nr2f1a* in control (ctrl), DEAB-treated, and RA-treated embryos at the 8s stage. (D-G) ISH for *nr2f1a* in the LPM of ctrl, RA, CHX, and RA+CHX treated embryos. (H) RT-qPCR of *nr2f1a* expression after RA and CHX treatments at the 3s stage. Asterisks in all graphs indicate statistically significant difference from controls with p<0.05.

**Fig 2:**
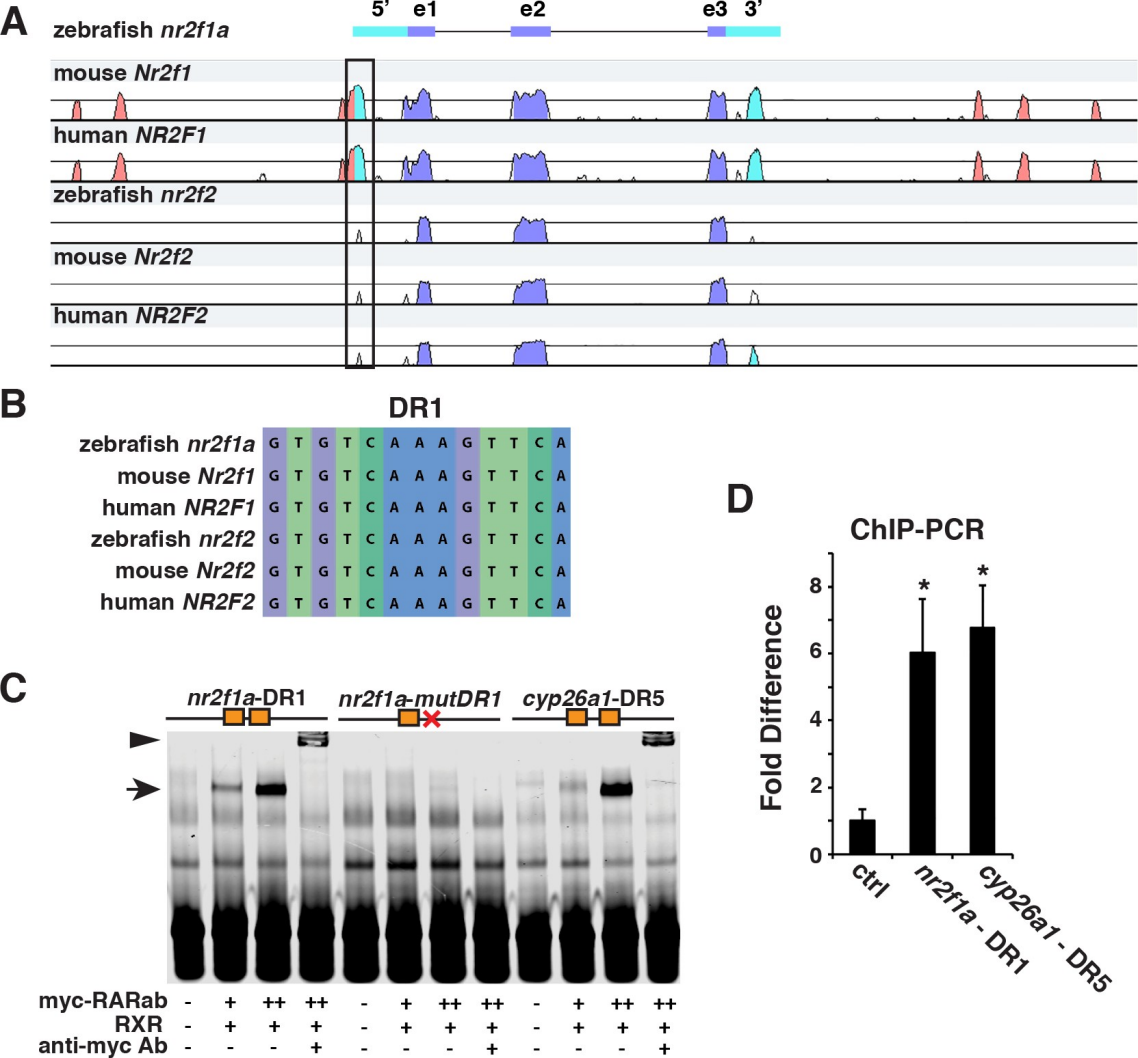
RARs bind a conserved DR1 site within the *nr2f1a* promoter. (A) mVISTA sequence alignment of 17kb coding and flanking regions for zebrafish, mouse, and human *NR2F1* and *NR2F2* genes. Box indicates region containing the conserved DR1 site. purple–conserved coding sequence, blue–conserved 5’- and 3’-UTRs, red–conserved sequences outside of the transcribed regions. (B) Conserved DR1 sequence in *NR2F* genes. (C) EMSA using probes for the *nr2f1a* - DR1, a mutated *nr2f1a* - DR1, and control *cyp26a1*—DR5 sites with increasing amounts of myc-tagged RARabv2 protein. Zebrafish RXRba protein was added to all samples as binding was not observed without RXR. (D) ChIP-qPCR of the *nr2f1a* DR1 site, negative control site, and a previously reported Cyp26a1 DR5 site (positive control) comparing the association of induced VP16-RARab from *hsp70l*:*EGFP-VP16-RARab* embryos to that in non-transgenic control sibling embryos at the 8s stage.

RA responsiveness of NR2F genes is conserved in chordates [28, 42–44]. We identified *nr2f1a* as an RA-responsive gene within the ALPM of zebrafish embryos (Fig 1A–1C), consistent with what other groups have described [28, 44]. However, the nature of this regulation has not been assessed. Furthermore, although RA signaling affects epigenetic modifiers that control the expression of *Nr2f1* in mammalian cells, a direct role for RA signaling has not been shown [30]. We found that RA treatment positively regulates *nr2f1a* expression after cycloheximide (CHX) treatment (Fig 1D–1H), implicating a direct transcriptional regulatory mechanism. To determine if there are putative RA response elements (RAREs) for RAR binding sites in the *nr2f1a* promoter region, we first performed a mVISTA alignment of zebrafish, mouse, and human *NR2F1* and *NR2F2* genomic sequences. We found a highly conserved region within the 5’-untranslated region (UTR) of *nr2f1a* (Fig 2A). Using the nuclear hormone receptor binding site prediction tool NHRscan in this region, we found a completely conserved direct repeat 1 (DR1) site [45–49] within the 5’-UTR of these genes (Fig 2B). While the location of this DR1 site is atypical, regulatory elements of other genes have been found to overlap with the 5’-UTR [50, 51]. Despite the conservation of these sites across phyla, the site was not present in the zebrafish paralog *nr2f1b*, which is not RA responsive [28]. Electrophoretic mobility shift assays (EMSAs) and chromatin immunoprecipitation-quantitative PCR (ChIP-qPCR) indicated that RARs can bind the *nr2f1a* DR1 *in vitro* and *in vivo* (Fig 2C and 2D). However, this site was not sufficient to respond to RA alone in luciferase assays (S1 Fig). Therefore, our results suggest RA directly regulates *nr2f1a* expression and may involve interactions with a conserved DR1 RARE, although this atypical site may not be responsive to RA through a canonical activation mechanism.

### *Nr2f1a* and *nr2f2* are redundantly required to restrict ventricular CM and promote posterior PM development

**Fig 3:**
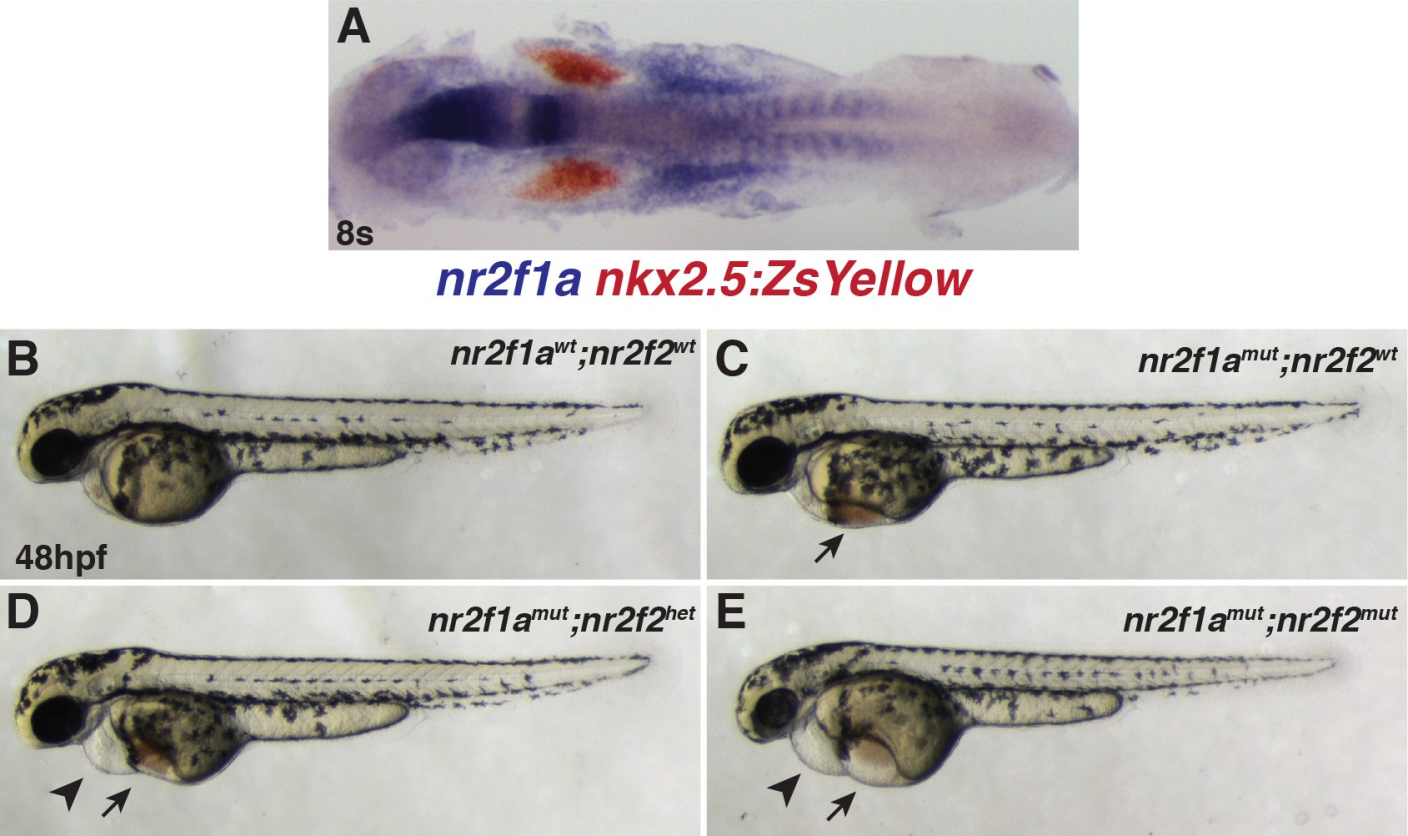
Loss of *nr2f2* alleles in *nr2f1a* mutants produces stronger overt cardiovascular defects. (A) Two-color ISH for *nr2f1a* (blue) and *nkx2*.*5*:*ZsYellow* (red) at the 8s stage. Embryo is flat-mounted with dorsal view and anterior left. (B-E) Lateral views of the *nr2f1a*^*wt*^; *nr2f2*^*wt*^, *nr2f1a*^*mut*^; *nr2f2*^*wt*^, *nr2f1a*^*mut*^; *nr2f2*^*het*^, and *nr2f1a*^*mut*^; *nr2f2*^*mut*^ embryos. n = 16 overtly WT embryos and n = 32 embryos with edema that were genotyped for the experiment shown. While embryos that have two WT alleles are shown, the other combinations of *nr2f1a* and *nr2f2* alleles, other than *nr2f1a*^*het*^; *nr2f2*^*mut*^ embryos, were indistinguishable from WT embryos at 48 hpf. 1 of 5 embryos that genotyped as *nr2f1a*^*het*^; *nr2f2*^*mut*^ embryos was indistinguishable from WT, while 4 of the 5 *nr2f1a*^*het*^; *nr2f2*^*mut*^ embryos displayed a very small amount of blood pooling on the yolk. However, cardiac defects were not found in these embryos. Arrows indicate edema and blood pooling on the yolk. Arrowheads indicated pericardial edema.

**Fig 4:**
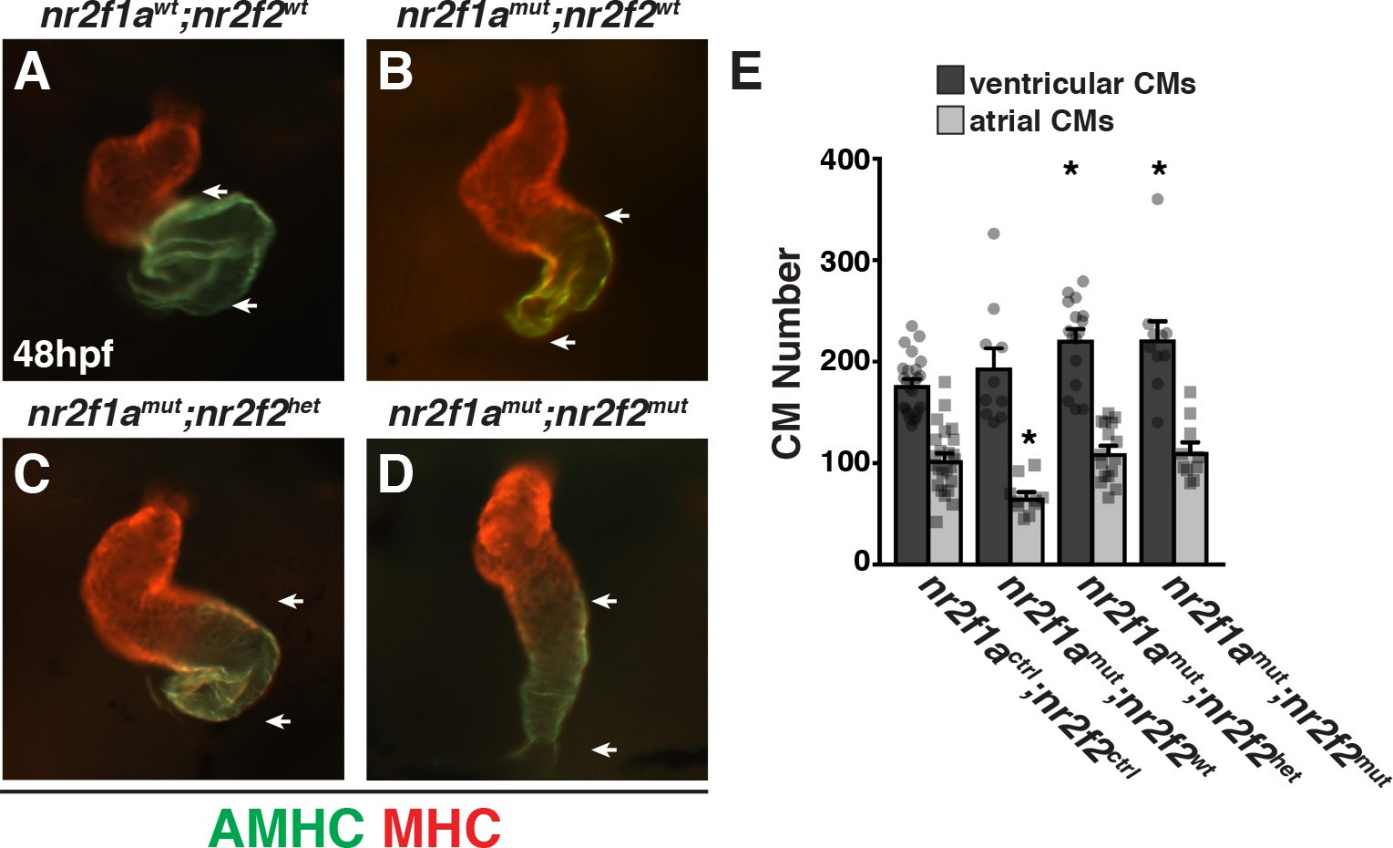
Nr2f1a functions redundantly with Nr2f2 to restrict ventricular CM number. (A-D) Frontal view of hearts from *nr2f1a*^*wt*^; *nr2f2*^*wt*^, *nr2f1a*^*mut*^; *nr2f2*^*wt*^, *nr2f1a*^*mut*^; *nr2f2*^*het*^, and *nr2f1a*^*mut*^; *nr2f2*^*mut*^ embryos at 48 hpf with immunohistochemistry (IHC). Atria (AMHC)—green. Ventricles (MHC)—red. (E) CM number from hearts of *nr2f1a*^*ctrl*^; *nr2f2*^*ctrl*^ (n = 24), *nr2f1a*^*mut*^; *nr2f2*^*wt*^ (n = 10), and *nr2f1a*^*mut*^; *nr2f2*^*het*^ (n = 16), and *nr2f1a*^*mut*^; *nr2f2*^*mut*^ (n = 10) embryos with the *myl7*:*h2afva-mCherry* transgene at 48 hpf. Although embryos WT for *nr2f1a* and *nr2f2* alleles are shown in all images for comparison to *nr2f1a*; *nr2f2* mutants, other allele combinations were indistinguishable from WT and pooled for data analysis. Therefore, *nr2f1a*^*ctrl*^; *nr2f2*^*ctrl*^ indicates analysis performed with the combination of *nr2f1a*^*wt*^; *nr2f2*^*wt*^; *nr2f1a*^*het*^; *nr2f2*^*wt*^, *nr2f1a*^*wt*^; *nr2f2*^*het*^, and *nr2f1a*^*het*^; *nr2f2*^*het*^ embryos.

**Fig 5:**
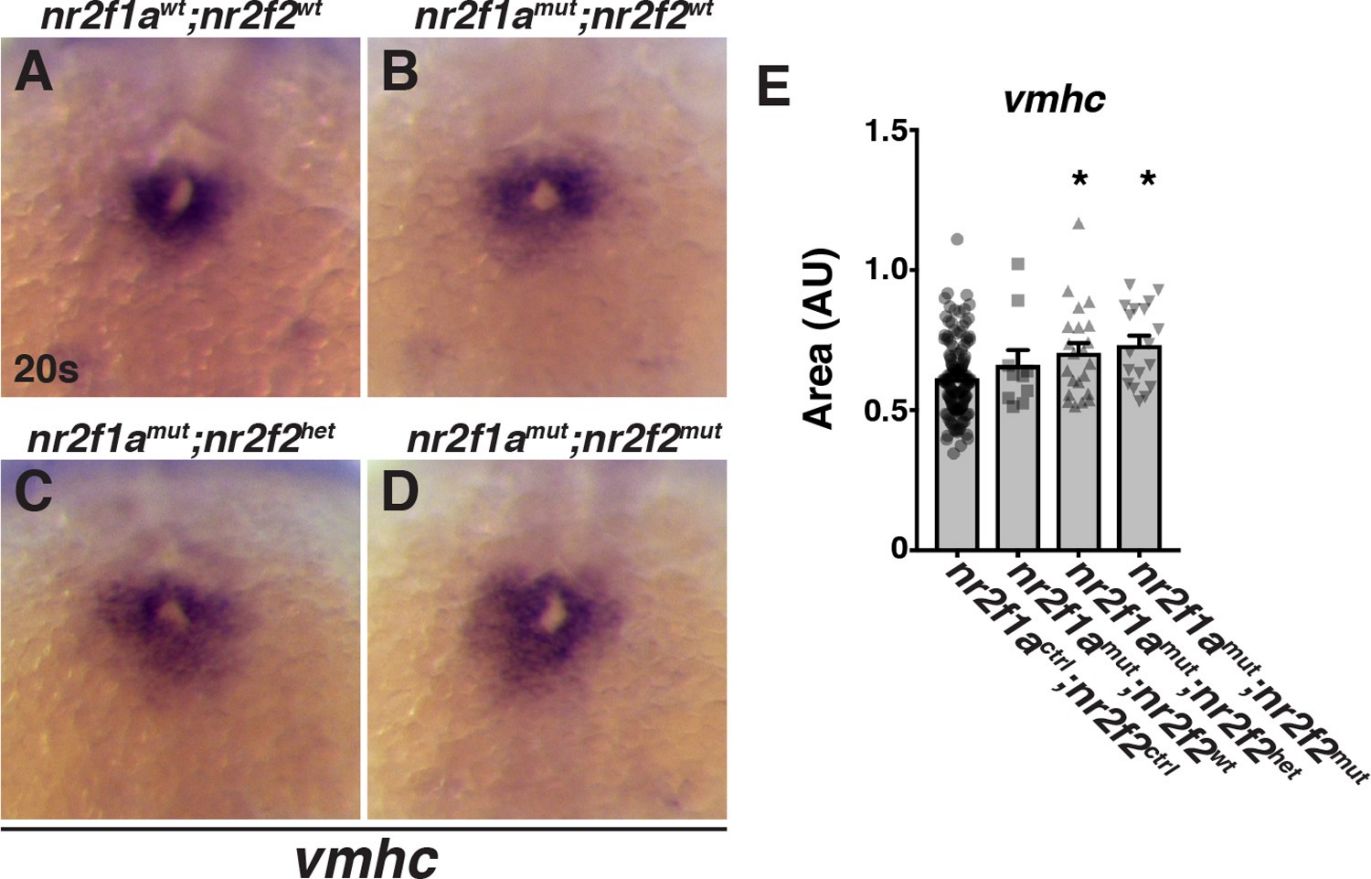
Nr2f1a and Nr2f2 redundantly restrict differentiation of ventricular CMs. (A-D) ISH for *vmhc* in *nr2f1a*^*wt*^; *nr2f2*^*wt*^, *nr2f1a*^*mut*^; *nr2f2*^*wt*^, *nr2f1a*^*mut*^; *nr2f2*^*het*^, and *nr2f1a*^*mut*^; *nr2f2*^*mut*^ embryos at the 20s stage. (E) Area measurements in arbitrary units (AU) of *vmhc* expression from *nr2f1a*^*ctrl*^; *nr2f2*^*ctrl*^ (n = 141), *nr2f1a*^*mut*^; *nr2f2*^*wt*^ (n = 8), *nr2f1a*^*mut*^; *nr2f2*^*het*^ (n = 22), and *nr2f1a*^*mut*^; *nr2f2*^*mut*^ (n = 18) embryos at the 20s stage.

Within the ALPM, zebrafish *nr2f1a* is expressed immediately posterior to cardiac progenitors during somitogenesis (Fig 3A). However, our recent study of *nr2f1a* mutants did not reveal requirements for Nr2f1a at these early developmental stages when the cardiac progenitor field is established [39]. Instead, we found that Nr2f1a is required to promote atrial CM differentiation at both the arterial and venous poles of the atrial chamber at subsequent stages of cardiogenesis, consistent with its expression specifically in atrial CMs within the developing cardiac tube [39]. Although zebrafish *nr2f2* mutants do not have overt cardiovascular defects through at least two weeks of development [37, 38], zebrafish *nr2f2* has low levels of expression within the ALPM during somitogenesis and is responsive to RA signaling (S2 Fig), albeit significantly less so than *nr2f1a* as has been previously shown [28]. Therefore, we wondered if Nr2f2 functions redundantly with Nr2f1a at earlier stages of development within the ALPM. Using established engineered zebrafish *nr2f2* deletion mutants [38], we found that loss of either one or both wild-type (WT) *nr2f2* alleles in *nr2f1a* mutant embryos resulted in overall progressively worse pericardial and yolk edemas coupled with blood pooling on the yolk compared to *nr2f1a* mutants alone (Fig 3B–3E). Similarly, we found that loss of *nr2f2* alleles in *nr2f1a* mutants produced hearts that were more dysmorphic and linear than *nr2f1a* mutant hearts alone (Fig 4A–4D). Despite the exacerbation of the cardiac dysmorphology in the compound *nr2f1a; nr2f2* mutants, we did not observe enhanced reduction of atrial chamber size or expression of AMHC, a marker of differentiated atrial CMs (Fig 4A–4D). Valve markers were also not further expanded with the loss of *nr2f2* alleles in *nr2f1a* mutants (S3 Fig), consistent with a unique role of Nr2f1a in limiting valve development [39]. Surprisingly, in contrast to *nr2f1a* mutants, which display a specific reduction in atrial CMs (Fig 4E; [39]), counting CMs with the *myl7*:*h2afva-mCherry* transgene [52] revealed that loss of one or both *nr2f2* alleles in *nr2f1a* mutants produced an equivalent increase in ventricular CMs without producing any deficit in atrial CMs (Fig 4E). Although we have found that the loss of atrial CMs is not due to early specification defects within the ALPM of *nr2f1a* mutants [39], we posited that the specific surplus of ventricular CMs in the *nr2f1a*; *nr2f2* mutants is due to an increase in ventricular CM specification at earlier stages of cardiogenesis because both *nr2f1a* and *nr2f2* are expressed within in the ALPM [28]. Consistent with this idea, in the double mutants we observed a modest expansion of the cardiac progenitor marker Nkx2.5 at the 16 somite (s) stage (S4 Fig) and the amount of differentiating ventricular CMs, indicated by *ventricular myosin heavy chain* (*vmhc*), was increased at the 20s stage (Fig 5A–5E). Furthermore, loss of both *nr2f1a* and *nr2f2* appeared to partially repress the ability of RA to inhibit *vmhc* expression (S5 Fig). Together, these data suggest that Nr2f1a and Nr2f2 function redundantly to restrict the number of differentiating ventricular CMs.

**Fig 6:**
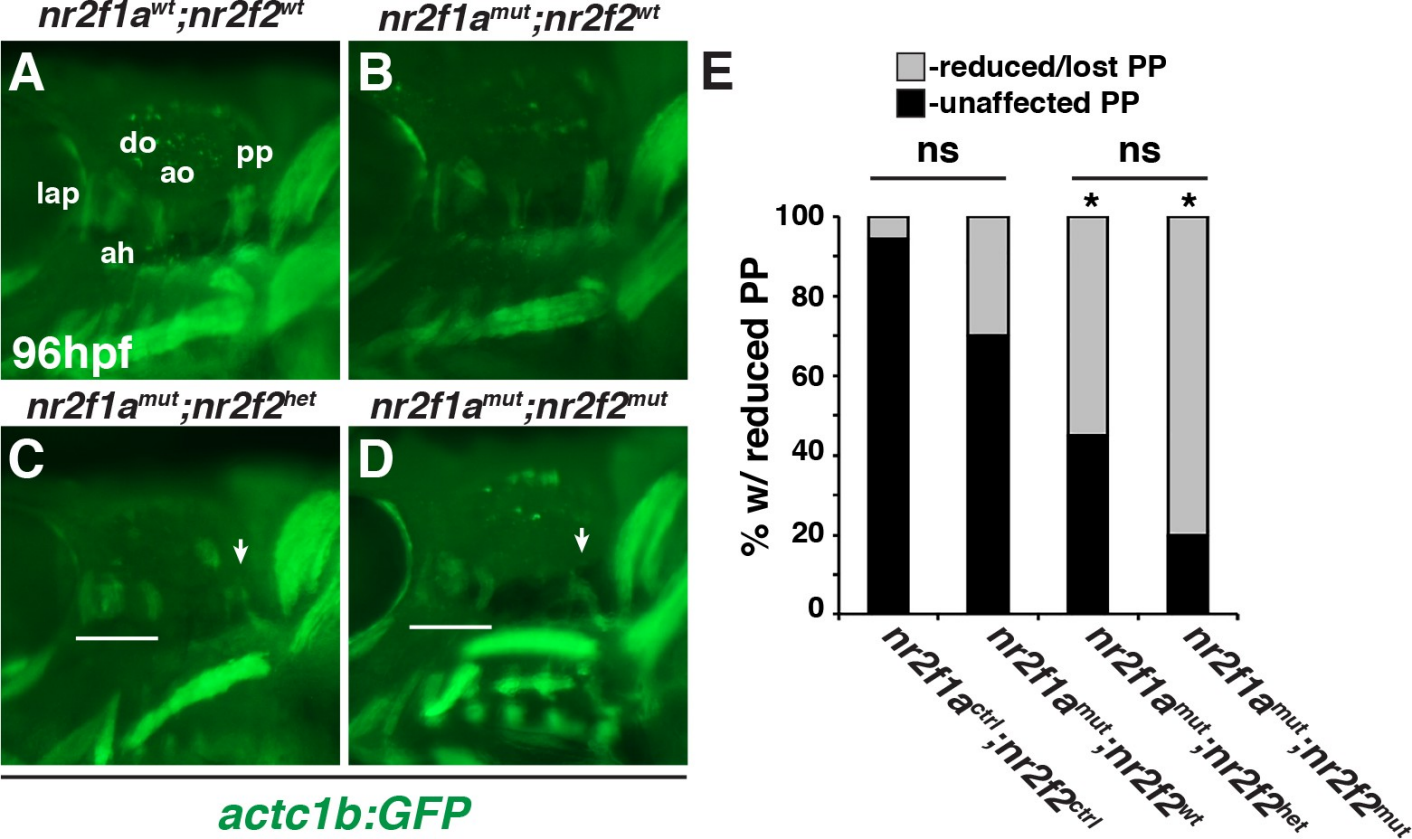
*Nr2f1a*; *nr2f2* mutants have deficiencies in their posterior PMs. (A-D) PMs in *nr2f1a*^*wt*^; *nr2f2*^*wt*^, *nr2f1a*^*mut*^; *nr2f2*^*wt*^, *nr2f1a*^*mut*^; *nr2f2*^*het*^, and *nr2f1a*^*mut*^; *nr2f2*^*mut*^ embryos with the *actc1b*:*GFP* transgene at 96 hpf. IHC was performed for GFP. Views are lateral with anterior to the left and dorsal up. Arrows in C and D indicate largely absent protractor pectoralis (pp). Brackets in C and D indicate 1^st^ and 2^nd^ arch PMs. ah–adductor hyoideus, ao–adductor operculi, do–dilator operculi, lap–levator arcus palitini. Muscle nomenclature used is from [55]. (E) Percentage of *nr2f1a*^*ctrl*^; *nr2f2*^*ctrl*^ (n = 18), *nr2f1a*^*mut*^; *nr2f2*^*wt*^ (n = 10), *nr2f1a*^*mut*^; *nr2f2*^*het*^ (n = 29), and *nr2f1a*^*mut*^;*nr2f2*^*mut*^ (n = 15) embryos with loss or malformed pp muscles at 96 hpf.

Previous analysis suggested that loss of RA signaling does not promote an increase in cardiac progenitor proliferation within the ALPM [53]. Consistent with this data, we did not find an increase in the number of proliferating Nkx2.5^+^ cells at the 16s stage in *nr2f1a*; *nr2f2* mutant embryos (S4 Fig). Thus, we postulated that the surplus ventricular CM progenitors in *nr2f1a*; *nr2f2* mutant embryos, which refers to *nr2f1a*^*mut*^ with either *nr2f2*^*het*^ or *nr2f2*^*mut*^ alleles, may be at the expense of an adjacent cell lineage. We reasoned that candidates were the pharyngeal arch arteries (PAAs) and PMs, since their progenitors intermingle with the cardiac progenitor population within the anterior mesoderm of zebrafish [5, 54]. We examined the posterior PAAs and PMs in *nr2f1a; nr2f2* mutants at 48 hpf and 96 hpf, developmental time points when these cells have respectively differentiated [55]. Interestingly, we did not detect defects in PAA number and morphology in *nr2f1a; nr2f2* mutant embryos carrying the *kdrl*:*EGFP* transgene (S6 Fig). However, in contrast to the PAAs, we found the posterior protractor pectoralis (pp), which is proposed to be a homolog of vertebrate neck muscles derived from the occipital LPM [56–60], was often lost or reduced in *nr2f1a*; *nr2f2* mutant embryos (Fig 6A–6E). Although not as dramatic, the anterior dorsal mandibular (1^st^) and hyoid (2^nd^) arch derived muscles were also often smaller and disorganized compared to WT and *nr2f1a* mutant siblings (Fig 6A–6D). A similar trend with respect to increased pp loss was observed at 75 hpf (S7 Fig). However, for the analysis of the compound mutants we focused on 96 hpf to ensure that any defects were not due to developmental delay. Together, these data suggest that Nr2f1a and Nr2f2 together are required to promote posterior PM development.

### Lineage tracing of *tcf21*^*+*^-derived progeny in *nr2f1a; nr2f2* mutant embryos

**Fig 7:**
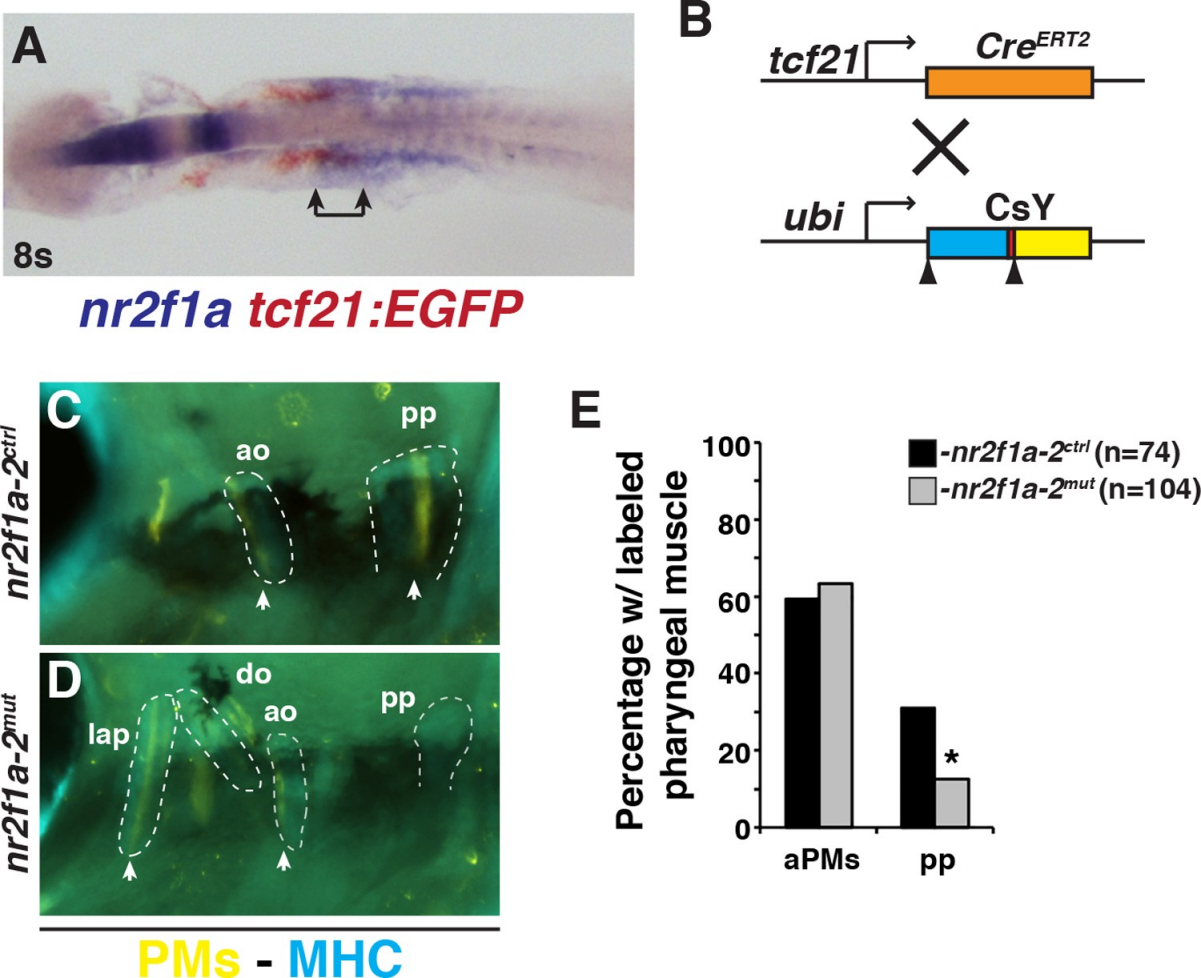
Tcf21^+^ progenitors less frequently contribute to the pp muscle in *nr2f1a-2* mutant embryos. (A) Two-color ISH of *nr2f1a* (blue) and *tcf21*:*EGFP* (red). Embryo is flat-mounted with anterior leftward. Bracket indicates region of overlap. (B) Schematic of *tcf21*:*Cre*^*ERT2*^ recombinase and ubiquitous Cre-mediated color-switch transgenic lines used. (C, D) PMs (arrowhead) labeled in *nr2f1a-2*^*ctrl*^ and *nr2f1a-2*^*mut*^ embryos with the *tcf21*:*Cre*^*ERT2*^; *ubi*:*CsY* transgenes. Labeled PMs–yellow. Muscles (MHC)–blue. Outlines indicate PMs with labeled skeletal muscles. While other cells were labeled within the pharyngeal region, they were not scored as skeletal muscle because they were not located within the muscles or had morphology consistent with skeletal muscle. View is lateral with anterior to the left and dorsal up. (E) Percentage of labeled PMs on each side of the *nr2f1a-*2^ctrl^ (n = 74) and *nr2f1a-2*^*mut*^ (n = 104) embryos. The (n) reflects the total number of sides examined, since labeling was not equivalent on both side of an embryo. aPMs—anterior pharyngeal muscles, pp—protractor pectoralis. 44/74 *nr2f1a-*2^ctrl^ and 66/104 *nr2f1a-2*^*mut*^ had muscle labeled in aPMs. 23/74 *nr2f1a-*2^ctrl^ and 13/104 *nr2f1a-2*^*mut*^ had muscle labeled in the pp. Fisher’s exact test was used to determine if there was a difference between the frequency of anterior and posterior PMs in *nr2f1a-*2^ctrl^ and *nr2f1a-2*^*mut*^ embryos.

Due to the inverse effects on ventricular CM and posterior PM development in the *nr2f1a*; *nr2f2* mutants, we sought to understand the relationship of these progenitors. Using two-color ISH to examine the expression of *nr2f1a* relative to *tbx1* and *tcf21*, we found that *nr2f1a* expression does not significantly overlap with *tbx1* (S8 Fig). However, *nr2f1a* and *tcf21* expression domains overlap in a caudal region of the ALPM (Fig 7A), interestingly, where lineage tracing has shown *tcf21*^*+*^ progeny give rise to CMs and posterior PM [5]. Despite the overlap in expression, *tcf21* expression was not affected in *nr2f1a*; *nr2f2* mutant embryos (S8 Fig). Since the *tcf21*^*+*^ progenitors are overtly specified properly in *nr2f1a*; *nr2f2* mutant embryos, we hypothesized that Nr2f proteins may affect a fate decision of progenitors within the posterior ALPM that can become ventricular and/or PM progenitors. To test this, we first used the inducible *tcf21*:*Cre*^*ERT2*^ transgene with the Cre-mediated color-switch line *ubi*:*LOXP-AmCyan-STOP-LOXP-ZsYellow (CsY)* to permanently label cells that have expressed *tcf21*^*+*^ (Fig 7B). For lineage tracing experiments, *nr2f1a* homozygous mutants (*nr2f1a*^*mut*^) coupled with *nr2f2* heterozygosity (*nr2f2*^*het*^) or *nr2f2* mutant homozygosity (*nr2f2*^*mut*^) were analyzed together (referred to as *nr2f1a-2*^*mut*^), because our data suggest loss of a single WT *nr2f2* allele in *nr2f1a* mutants produces similar ventricular CM and PM defects as loss of both WT alleles in *nr2f1a* mutants. Consistent with what has been reported [5], we found that tamoxifen treatment of embryos containing both transgenes produced labeling of skeletal muscle within the PMs (Fig 7C–7E). Although we did not find a decrease in the frequency of labeled anterior PMs within the 1^st^ and 2^nd^ arches, we found a decrease in the frequency of contribution to the pp in the *nr2f1a-2*^*mut*^ embryos (Fig 7E), supporting that Nr2f proteins promote the differentiation of skeletal muscle within the pp.

**Fig 8:**
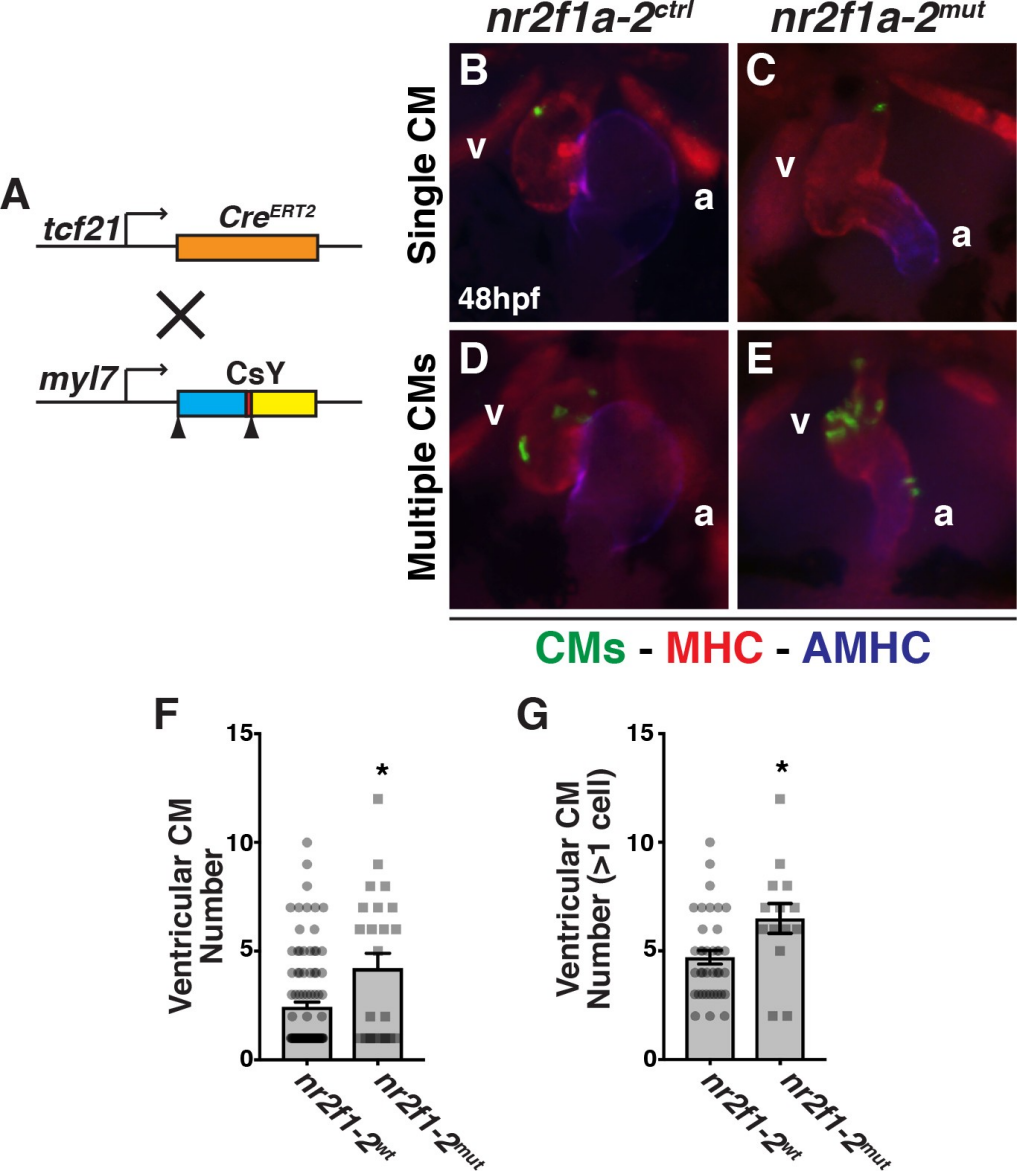
Nr2f proteins are required to limit ventricular CMs. (A) Schematic of *tcf21*:*Cre*^*ERT2*^ recombinase and *myl7* promoter driven Cre-mediated color-switch transgenic lines used. (B-E) Hearts with labeled CMs in *nr2f1a-*2^ctrl^ and *nr2f1a-2*^*mut*^ embryos carrying the *tcf21*:*Cre*^*ERT2*^; *myl7*:*CsY* transgenes. Labeled CMs–green. Cardiac muscles (MHC)–red. Atrium (AMHC)–blue. Images are frontal views. (F) Mean number of ventricular CMs labeled in *nr2f1a-*2^wt^ (n = 98) and *nr2f1a-2*^*mut*^ (n = 24) embryos. (G) Mean number of ventricular CMs labeled when more than one labeled ventricular CM was found in *nr2f1a-*2^wt^ (n = 38) and *nr2f1a-2*^*mut*^ (n = 14) embryos.

We then reasoned that if Nr2f proteins are influencing a fate decision of ventricular and PM progenitors, *tcf21*^+^ progenitors should become ventricular CMs at an increased frequency in *nr2f1a; nr2f2* mutant embryos. While we found that using *tcf21*:*Cre*^*ERT2*^; *ubi*:*CsY* labeled a few CMs, the expression was not as robust as for the PM. Therefore, we used the *myl7*:*CsY* transgene in combination with the *tcf21*:*Cre*^*ERT2*^ transgene to specifically and permanently label CMs derived from *tcf21*^*+*^ progenitors (Fig 8A–8E). Examining labeled ventricular CMs, we found a trend where *nr2f1a-2*^*mut*^ embryos have an increase in the number of embryos with >1 *tcf21*^*+*^-derived ventricular CM labeled compared to control embryos (S9 Fig). Importantly, overall, *nr2f1a-2*^*mut*^ embryos on average have about twice as many *tcf21*^*+*^-derived ventricular CMs compared to WT sibling embryos (Fig 8F). Furthermore, there were increased number of labeled ventricular CMs found in *nr2f1a-2*^*mut*^ embryos when just examining the pool of embryos that had >1 ventricular CM labeled (Fig 8G), further supporting an increase in the frequency and number of *tcf21*^*+*^-derived ventricular CMs contributing to the ventricles in *nr2f1a-2*^*mut*^ embryos. While atrial CMs were also labeled, their labeling was infrequent compared to labeling of ventricular CMs (S9 Fig). We did not find a statistical difference in the frequency or average number of atrial CMs labeled within the populations (S9 Fig). Together, our lineage tracing of *tcf21*^*+*^ progenitors demonstrates that a greater number of their progeny give rise to ventricular CMs in *nr2f1a-2*^*mut*^ embryos, while fewer give rise to the pp.

## Discussion

Previous studies have demonstrated that RA signaling is necessary to limit cardiac specification and promote PM development [22, 61]. With respect to heart development, early RA signaling restricts the posterior border of atrial and ventricular progenitors within the ALPM [25]. Despite similar effects on both cardiac cell types, mechanisms restricting atrial CMs and ventricular CMs downstream of RA signaling appear to be temporally distinct [25]. The present study suggests that Nr2f1a and Nr2f2 function redundantly downstream of RA signaling within the ALPM to regulate these converse effects on ventricular CM and PM specification. While there are numerous similarities between our observations in comparison to RA signaling-deficient embryos [22, 25], it is worth recognizing that the heart and PM defects found in *nr2f1a*; *nr2f2* mutant embryos are less severe than what is typically found with RA signaling-deficient embryos. Therefore, we hypothesize that these Nr2f transcription factors likely are part of a larger RA-responsive gene network, including Hox genes and Fgf signaling, that contributes to this allocation of progenitors within the ALPM.

Recent work examining Nr2f proteins in cranial neural crest that generate the anterior jaw has suggested significant redundancy with Nr2f1b and Nr2f5 in that developmental context [38]. However, we have not found any evidence of redundancy or genetic interactions with Nr2f1b and/or Nr2f5 in regulating heart development. For example, unlike what is observed with *nr2f1a* and *nr2f2*, *nr2f1a*^*+/-*^*;nr2f5*^*+/-*^ intercrosses produce ~25% (16/56) mutant embryos that are indistinguishable from *nr2f1a* mutant embryos with respect to the heart and blood pooling. We also have not found evidence for compensatory expression of any *nr2f* genes in the *nr2f1a* mutants (S10 Fig). Additionally, we have not found defects in neural crest markers in the *nr2f1a*; *nr2f2* mutants (S8 Fig), suggesting loss of the pp is not secondary to neural crest defects. Our recent work suggests that Nr2f1a alone functions to promote atrial CM differentiation as the heart elongates and atrial CMs mature [39], which is after it first appears in the ALPM. Here, we demonstrate that the change in the number of atrial CMs is not exacerbated in the *nr2f1a*; *nr2f2* mutant embryos compared to *nr2f1a* single mutants. Instead, the number of atrial CMs is increased relative to *nr2f1a* single mutants, despite a similar overt reduction in atrial chamber size and lack of AVC, and equivalent to the number found in control embryos. We posit that these differential effects on the production of atrial CMs are because Nr2f proteins restrict the posterior extent of both atrial and ventricular progenitor fields within the ALPM and that a deficit in differentiating atrial CMs is not observed because the earlier requirements limiting the cardiac progenitor field offset the later requirements promoting atrial CM differentiation.

NR2Fs are conserved regulators of atrial chamber development in vertebrates. Zebrafish *nr2f1a* mutants and mouse global *Nr2f2* KOs present smaller atria [35, 39], while conditional *Nr2f*2 KO in the heart at later stages suggests a role in maintenance of atrial CM identity [36]. NR2F2 is required for atrial CM differentiation in human iPSCs [32]. Given these conserved requirements, it is interesting to compare the phenotypes of the *nr2f1a*; *nr2f2* double mutants to the variability and severity of CHDs associated with *NR2F2* lesions in humans. It has been proposed there is a direct correlation between the severity of CHDs and types of lesions impacting NR2F2 function [41]. Specifically, nonsense mutations proposed to be more damaging and resulting in significant loss of NR2F2 predominantly are associated with LVOTO, while missense mutations proposed to be less damaging are associated with ASDs and AVSDs [41]. The variable CHDs affecting the arterial pole of the ventricle and the atrial chamber are highly reminiscent of the chamber-specific defects we observe in *nr2f1a*; *nr2f2* double mutants compared to single *nr2f1a* mutants, which overtly affect the production of ventricular CMs and atrial CMs, respectively. Together, these data support the hypothesis that levels of total Nr2f dosage differentially affect chamber-specific cardiogenic processes within the vertebrate heart. Moreover, we propose that greater loss of NR2F transcription factors, through more damaging alleles or genetic loss, produces ventricular chamber defects due to earlier developmental requirements within the ALPM, while ASDs or AVSDs may occur due to a more modest loss of total NR2F signaling that is required at later stages of atrial CM differentiation. Thus, our studies offer a working model to explain the molecular etiology of congenital LVOTO and ASDs/AVSDs associated with *NR2F2* mutations in humans.

While NR2F proteins have been studied in numerous development contexts, significant analysis of the requirements for Nr2fs in skeletal muscle have not been reported. Virtually all the Nr2f proteins are expressed in the somites of zebrafish [28]. Interestingly, in mice *Nr2f2* is broadly expressed in skeletal muscle, including the somites and the cranial muscles [34, 41]. Limb-specific *Nr2f2* KOs indicate it is required for limb muscle development [34] and mechanistically there is evidence that Nr2f2 can compete with myoD in muscle differentiation [62]. Therefore, there is precedence for Nr2f2 functions in somite-derived skeletal muscle, but requirements in PM development have not been reported. It is interesting to note that cranio-facial defects have been associated with genetic deficiencies that affect both *NR2F1* and *NR2F2* in humans [40, 63, 64]. In two independent cases, similar-sized deletions that eliminate *NR2F2* were associated with cranial abnormalities as well as ASDs [40, 64]. However, overt cranio-facial defects similar to those found in the deficiencies were not reported in patients found to have specific mutations that affect *NR2F2* and are associated with CHDs [41]. Therefore, although specific defects in craniofacial muscle were not reported, there is precedence for an association between *NR2F* gene loss and both craniofacial and CHDs in humans.

Recent clonal analysis in mice has suggested there are common cardio-pharyngeal progenitors that contribute progeny to the neck muscles, the arterial pole and atria that are distinct from other cardio-pharyngeal populations of the SHF [8]. Our data are also consistent with a close association of ventricular outflow tract and pharyngeal neck muscle progenitors and a distinction from other SHF progenitors, which arise more anteriorly [65, 66]. Specifically, while anterior dorsal 1^st^ and 2^nd^ arch muscles are reduced, we predominantly find that *nr2f1a*; *nr2f2* embryos lose the posterior pp muscle (cucullaris), which has been proposed to be homologous to the ALPM-derived trapezius neck muscles in mammals [56–60]. Therefore, reminiscent of the recent retrospective clonal analysis in mice [8], these results hint at the existence of a distinct posterior progenitor population with cardiac and PM potential that does not correspond to the anterior SHF.

Given the existence of bi-potent cardio-pharyngeal progenitors in mice and *Ciona* [6–8, 19, 20], one interpretation of our results is that RA signaling and consequently Nr2f proteins, at least in part, act on bi-potent cardio-pharyngeal progenitors. Although it is clear from the retrospective clonal lineage analysis in mice that there are multiple populations of bi-potent cardio-pharyngeal progenitors, these populations are rare and only found from examination of large sample sizes [6–8]. While zebrafish *tcf21*^*+*^, as well as *nkx2*.*5*^*+*^ progenitors, can give rise to ventricular CMs and PMs [5, 67], it is not yet clear whether there are bi-potent progenitors with cardiac and PM potential. Although the defects we observe in ventricular CM and PM development of *nr2f1a*; *nr2f2* mutant embryos are less dramatic than with loss of RA signaling, in neither case are the defects subtle enough that a very rare population of bi-potent progenitors is likely being affected. Instead, we favor a model where there is a larger population of progenitors within the ALPM that have the potential to become either ventricular CMs and PM, with signals such as Nr2f proteins functioning downstream of RA signaling to influence their allocation into one of these populations.

Overall, our study provides valuable insight into the requirements of Nr2f genes in vertebrate cardiac and cranial muscle development. These studies may help us to further understand molecular and genetic etiology controlling phenotypic variability of CHDs as well as developmental syndromes that have congenital malformations concomitantly affecting the heart, head, and neck muscles in humans.

## Methods

### Ethics statement

All zebrafish husbandry and experiments were performed in accordance with protocols approved by the Institutional Animal Care and Use Committee (IACUC) of Cincinnati Children's Hospital Medical Center.

### Zebrafish line and maintenance

Adult zebrafish were raised and maintained under standard laboratory conditions. Transgenic lines used were: *Tg(kdrl*:*nlsEGFP)*^*ubs1*^ [68], *Tg(kdrl*:*EGFP*)^*la116*^, *TgBAC(−36nkx2*.*5*:*ZsYellow)*^*fb7*^ [69], *Tg(actc1b*:*GFP)*^*zf13*^ [70], *Tg(tcf21*:*nucEGFP)*^*pd41*^ [71], *Tg(tcf21*:*CreER*^*T2*^*)*^*pd42*^ [72], *Tg(ubi*:*LOXP-AmCyan-STOP-LOXP-ZsYellow)*^*fb5*^ [69], *Tg(myl7*: *LOXP-AmCyan-STOP-LOXP-ZsYellow)*^*fb2*^ [69], *Tg(myl7*:*h2afva-mCherry)*^*sd12*^ [52] and *Tg(hsp70l*:*EGFP-VP16-RAR-)*^*c1004*^ [73]. Mutant alleles used were: *nr2f1a*^*el512*^ and *nr2f2*^*el506*^ [38, 39].

### Whole mount ISH and area measurements

Single and two-color whole mount ISH were performed using NBT/BCIP (Roche) and INT/BCIP (Roche), as previously reported [74]. Digoxygenin- and fluorescein-labeled anti-sense RNA probes for *zsyellow* (ZDB-EFG-110824-1), *egfp* (ZDB-EFG-070117-1), *nr2f1a* (ZDB-GENE-980526-115), *nr2f2* (ZDB-GENE-990415-252), *vmhc* (ZDB-GENE-991123-5), *nkx2*.*5* (ZDB-GENE-980526-321), *dlx2a* (ZDB-GENE-980526-212), *tbx1* (ZDB-GENE-030805-5), *tcf21*(ZDB-GENE-051113-88), and *klf2a* (ZDB-GENE-011109-1) were used. Area measurements were performed using ImageJ.

### Analysis of Nr2f loci and promoters

Sequences for zebrafish, mouse, and human *Nr2f* genes plus a 10kb region 5’ and 3’ to the genes were taken from Ensembl (ensembl.org) and aligned using mVista (http://genome.lbl.gov/vista/mvista/submit.shtml). Locations, excluding exons, in which there was over 50% conservation between any of the sequences were analyzed for the presence of RARs. Conserved sequences were input into NHRscan (http://www.cisreg.ca/cgi-bin/NHR-scan/nhr_scan.cgi) to identify potential RAR binding sites.

### RT-qPCR

Total RNA isolation and RT-qPCR was performed using previously reported methods [75]. Briefly, whole embryo RNA was obtained from groups of 30 embryos using Trizol (Ambion) and Purelink RNA Microkit (Invitrogen). cDNA was synthesized using 1μg total RNA and the ThermoScript Reverse Transcriptase kit (Invitrogen). RT-qPCR was performed using Power SYBR Green PCR Master Mix (Applied Biosystems) in a BioRad CFX-96 PCR machine. Expression levels were standardized to *β-actin* expression and data were analyzed using the 2^-ΔΔCT^ Livak Method. All experiments were performed in triplicate. Primer sequences for *β-actin* were reported previously [73, 75]. All primer sequences used for RT-qPCR are in the S1 Table.

### Drug treatments

All drug treatments were administered to embryos in 2 mL of blue water with drug at specified concentrations in a glass vial with 25–30 embryos/vial at 28.5°C. For analysis of *nr2f1a* and *nr2f2* expression, embryos were treated with CHX (10 μM, Sigma 48591), RA (1 μM, Sigma R2625), and DEAB (10 μM, Sigma D86256) at tailbud stage for 1 hour. For analysis of *vmhc* expression in *nr2f1a*; *nr2f2* mutants, embryos were treated with 0.05 μM RA at the 3s stage until the 20s stage. Drugs were washed out 3X with embryo water then the embryos were fixed in 4% formaldehyde for analysis. *Vmhc*-stained embryos were genotyped following imaging. Tamoxifen (10 μM, Sigma H7904) was administered in 30 mL of blue water with 0.003% PTU in petri dishes to embryos at 30%-50% epiboly until embryos were analyzed or through 2 days of development.

### ChIP-qPCR

ChIP-qPCR was performed essentially as previously reported [75]. Hemizygous *Tg(hsp70l*:*VP16-RAR*:*EGFP)*^*c1004*^ adults were crossed to WT adult zebrafish. The resulting embryos were collected at tailbud stage and heat-shocked at 37°C for 30 minutes. Transgenic embryos were sorted from their non-transgenic control siblings by the presence of GFP. Embryos (n = 100) were dechorionated and fixed in 1% formaldehyde 2 hours after heat-shock. Cells were lysed by gentle pipetting in cell lysis buffer. Nuclei were lysed and DNA was sheered by sonication with glass beads to 200-600bp fragments. Dynabeads (Invitrogen) were used to pull down GFP tagged proteins with ChIP-grade polyclonal anti-GFP antibody (Abcam ab290) per manufactures instructions. Samples were de-crosslinked and qPCR was used to quantify the fold difference in enrichment of the DR1 RARE in the *nr2f1a* promoter and the known DR5 RARE in the Cyp26a1 promoter as compared to a *nr2f1a* promoter region not containing a RARE. Expression levels were standardized to the no antibody control signal and data were analyzed using the 2^-ΔΔCT^ Livak Method. Primer sequences for *cyp26a1* ChIP-PCR were reported previously [76]. Primer sequences for *nr2f1a* DR1 ChIP-PCR and control are indicated in S1 Table.

### EMSA

EMSA was performed essentially as previously reported [77]. Oligonucleotides were designed containing the *nr2f1a* DR1 site (GTGTCAAAGTTCA), the *nr2f1a* DR1 site with a targeted mutation in the second half site of the DR1 abolishing the direct repeat (GTGTCAAAGTCAT), and a previously reported Cyp26a1 DR5 site [76]. A complementary oligonucleotide was designed with a 5’ LI-COR IRDye 700 (IDT). The oligonucleotides were annealed and the ends filled with Klenow (New England Biolabs). Zebrafish *myc-rarab* was in the pCS2+MT. Zebrafish RXRba was cloned into pCS2p+. Proteins for EMSA were made using the TnT SP6 Quick Coupled Transcription/Translation System (Promega). Protein samples were gently mixed with LI-COR tagged probes and incubated at room temperature for 20 minutes. 4% polyacrylamide gels were run for 2 hours at 150 V. Gels were imaged using an Odyssey CLx LI-COR imager.

### Zebrafish IHC and CM counts

Embryos were fixed for 1 hour at room temperature in 1% formaldehyde in PBS in 3 ml glass vials. Embryos were washed 1X in PBS and then 2X in 0.2% saponin/1X PBS, followed by blocking in 0.2% saponin/0.5%sheep serum/1X PBS (Saponin blocking solution) for one hour. AMHC (S46) and MHC (sarcomeric myosin; MF20) primary antibodies (Developmental Studies Hybridoma Bank) were incubated at 1:10 in Saponin blocking solution. Rabbit polyclonal DsRed antibody (Clontech), to detect mCherry, and Living colors anti-RCFP (Clontech), to detect ZsYellow, were used at a 1:1000 dilution. Rabbit anti-GFP (Abcam) was used at 1:500. Rabbit anti-Nkx2.5 (Gene Tex) was used at 1:250. Mouse anti-pHH3 (Abcam) was used at 1:1000. All secondary antibodies were used at dilutions of 1:100. Antibody information is also listed in S2 Table. Cell counts were performed by gently flattening embryos under a coverslip and counting the fluorescent nuclei in each chamber. For all imaging except Nkx2.5/pHH3, embryos were imaged using a Zeiss M2BioV12 Stereo microscope. For Nkx2.5/pHH3, embryos were post-fixed in 2% formaldehyde/1X PBS for two hours and mounted in 1% low-melt agar on 2% agar plates. Images of one side of the embryo were taken using a Nikon A1R Multiphoton Upright Confocal Microscope with a 16X water immersion objective. 200 μm optical sections were taken with the resonance scanner.

### Luciferase assays

The promoter fragments for both reporters used were cloned into the Kpn and HindIII sites of the pGL3 (Promega) multiple cloning site. The *DR1-ef1a* construct contains 165 base pairs (bp) of the *nr2f1a* promoter and 5’UTR adjacent to 193 bp of a minimal *elongation factor 1a* (*ef1a*) promoter (green). The *nr2f1a-DR1* construct contains 371 bp that include the promoter and 5’UTR containing the conserved DR1 site. The pGL3-12XRARE-tk vector and dual luciferase assays were reported and performed in HEK293 cells as described previously [78].

### Statistical analysis

To compare two groups, we performed a Student’s *t-*test or Mann-Whitney test. To compare 3 or more conditions are different, we performed ANOVA analysis. To determine if two proportions were statistically distinct we performed a Chi-squared test or Fisher’s exact test. Statistical analysis was performed using GraphPad Prism. A *p* value < 0.05 was considered statistically significant for all analysis.

## Supporting information

S1 Fig *In vitro* reporter assay for RA responsiveness of *nr2f1a* DR1 site. (A) Schematic of the two constructs placed into pGL3. The *pGL3-DR1-ef1a* construct has nucleotides -60 of the *nr2f1a* promoter (blue) through +105 of 5’UTR (gray), which includes the including the DR1 site (yellow box) cloned adjacent to a minimal *elongation factor 1a* (*ef1a*) promoter (green) (358 bp). The *pGL3-nr2f1a-DR1* construct contains nucleotides -266 through +105 (371 bp) of the promoter and 5’UTR containing the conserved DR1 site. Blue indicates *nr2f1a* promoter sequences. Red boxes indicate predicted TATA boxes. (B) Luciferase assays testing RA responsiveness in HEK 293 cells. FF–firefly luciferase. RL—renilla luciferase. The previously reported *pGL3-12XRARE-tk* plasmid [78] was used as a positive control.
(TIF)

S2 Fig *Nr2f2* expression at the 10 somite stage. (A-C) *Nr2f2* expression in the ALPM of control, DEAB-treated, and RA-treated embryos. View is dorsal with anterior left. Arrows indicated anterior and posterior limits of expression in control and RA-treated embryos.
(TIF)

S3 Fig Atrioventricular valve marker defects are not exacerbated with loss of *nr2f* gene alleles. (A-D) ISH for the endocardial atrioventricular canal marker *klf2a*. Frontal views of hearts in *nr2f1a*^*wt*^; *nr2f2*^*wt*^, *nr2f1a*^*mut*^; *nr2f2*^*wt*^, *nr2f1a*^*mut*^; *nr2f2*^*het*^, and *nr2f1a*^*mut*^; *nr2f2*^*mut*^ embryos. v–ventricle. a–atrium. Arrows indicate the length of *klf2a* expression within the hearts.
(TIF)

S4 Fig *Nkx2*.*5* is expanded in *nr2f1a*; *nr2f2* double mutant embryos. (A-D) ISH for the cardiac progenitor marker *nkx2*.*5* in *nr2f1a*^*wt*^; *nr2f2*^*wt*^, *nr2f1a*^*mut*^; *nr2f2*^*wt*^, *nr2f1a*^*mut*^; *nr2f2*^*het*^, and *nr2f1a*^*mut*^; *nr2f2*^*mut*^ embryos at the 16s stage. Dorsal view with anterior up. 160 embryos were examined with ≥9 embryos examined for each condition. Although we observed a trend in the expansion of *nkx2*.*5* expression when assaying area of expression similar to *vmhc*, due to inherent variability in *nkx2*.*5* expression and the low numbers of embryos, it was not statistically significant. (E,F) IHC for Nkx2.5 and pHH3 in *nr2f1a*^*wt*^; *nr2f2*^*het*^ and *nr2f1a*^*mut*^; *nr2f2*^*mut*^ embryos at the 16s stage. Confocal images of the ventro-lateral side of the embryo. Dorsal is right and anterior up. A single side of each embryo was used for analysis. (G) Number of Nkx2.5^+^ cells in control and *nr2f1a*; *nr2f2* mutant embryos. (H) Percentage of pHH3^+^/Nkx2.5^+^ in control and *nr2f1a*; *nr2f2* mutant embryos. For quantification of Nkx2.5^+^ and pHH3^+^/Nkx2.5^+^ cells, *nr2f1a* homozygous mutants (*nr2f1a*^*mut*^) coupled with *nr2f2* heterozygosity (*nr2f2*^*het*^) or *nr2f2* mutant homozygosity (*nr2f2*^*mut*^) were analyzed together (referred to as *nr2f1a-2*^*mut*^), because our data suggest loss of a single WT *nr2f2* allele in *nr2f1a* mutants produces a similar increase in ventricular CMs as double mutants. *Nr2f1a-*2^ctrl^ includes any combination of *nr2f1a* and *nr2f2* WT and heterozygous alleles. *nr2f1a-*2^ctrl^ (n = 23) and *nr2f1a-2*^*mut*^ (n = 9) for G and H.
(TIF)

S5 Fig RA-induced repression of *vmhc* expression is sensitized to loss of *nr2f1a* and *nr2f2*. (A-C) ISH for *vmhc* in control (untreated), RA-treated *nr2f1a*^*wt*^; *nr2f2*^*het*^, and RA-treated *nr2f1a*^*mut*^; *nr2f2*^*mut*^ embryos at the 20s stage. Control embryos were not genotyped. (D) Percentage of embryos with the genotypes found that lacked *vmhc* expression (n = 16) or had *vmhc* expression (n = 16). Although a RA-treated *nr2f1a*^*wt*^; *nr2f2*^*het*^ is shown in B, *nr2f1a-*2^ctrl^ includes any combination of *nr2f1a* and *nr2f2* WT and heterozygous alleles. Fisher’s exact test was used to compare the frequency of embryos with two *nr2f1a*^*mut*^ alleles found in each condition.
(TIF)

S6 Fig The PAAs are unaffected in *nr2f1a-2* mutant embryos. (A,B) PAAs in *nr2f1a-2*^*ctrl*^ and *nr2f1a-2*^*mut*^ embryos. Numbers indicated arches. Anterior is to the right.
(TIF)

S7 Fig The pp is reduced in *nr2f1a*; *nr2f2* mutant embryos. (A-D) PMs in *nr2f1a*^*wt*^; *nr2f2*^*wt*^, *nr2f1a*^*mut*^; *nr2f2*^*wt*^, *nr2f1a*^*mut*^; *nr2f2*^*het*^, and *nr2f1a*^*mut*^; *nr2f2*^*mut*^ embryos at 75 hpf. Views are lateral with anterior to the left and dorsal up. (E) Percentage of *nr2f1a*^*ctrl*^; *nr2f2*^*ctrl*^ (n = 7), *nr2f1a*^*mut*^; *nr2f2*^*wt*^ (n = 16), *nr2f1a*^*mut*^; *nr2f2*^*het*^ (n = 28), and *nr2f1a*^*mut*^; *nr2f2*^*mut*^ (n = 28) embryos with loss of posterior and malformed PMs at 75 hpf.
(TIF)

S8 Fig PM progenitor and cranial neural crest markers are not affected in *nr2f1a; nr2f2* mutant embryos. (A) ISH for *tbx1* (red) and *nr2f1a* (blue) in the ALPM of an embryo at the 8s stage. Image is a dorsal view with anterior rightward of a flat-mounted embryo. (B-E) ISH for *tcf21* in the ALPM of *nr2f1a*^*wt*^; *nr2f2*^*wt*^, *nr2f1a*^*mut*^; *nr2f2*^*wt*^, *nr2f1a*^*mut*^; *nr2f2*^*het*^, and *nr2f1a*^*mut*^; *nr2f2*^*mut*^ embryos at the 18s stage. (F-I) ISH for the neural crest marker *dlx2a* in *nr2f1a*^*wt*^; *nr2f2*^*wt*^, *nr2f1a*^*mut*^; *nr2f2*^*wt*^, *nr2f1a*^*mut*^; *nr2f2*^*het*^, and *nr2f1a*^*mut*^; *nr2f2*^*mut*^ embryos at the 18s stage. For B-I, views are dorsal with anterior up.
(TIF)

S9 Fig Frequency of labeled CMs in *tcf21*:*CreERT2*; *myl7*:*CSY* embryos. (A) Percentage of embryos with 1 and >1 ventricular CM. (B) Percentage of embryos with labeled CMs that had labeled atrial CMs. (C) Mean number of labeled atrial CMs in *nr2f1a-2*^*ctrl*^ and *nr2f1a-2*^*mut*^ embryos.
(TIF)

S10 Fig *Nr2f* gene expression in *nr2f1a* mutants. RT-qPCR for *nr2f1b*, *nr2f2*, *nr2f5*, *nr2f6a*, and *nr2f6b* in *nr2f1a* mutants at 48 hpf does not show compensatory expression.
(TIF)

S1 Table Primers sequences. (DOCX)

S2 Table Antibodies used. (DOCX)
