# Supplementary figures and images for: Nr2f-dependent allocation of ventricular cardiomyocyte and pharyngeal muscle progenitors
Source: PLoS Genet. 2019 Feb 5;15(2):e1007962. doi: 10.1371/journal.pgen.1007962 (PMC6377147; doi:10.1371/journal.pgen.1007962)

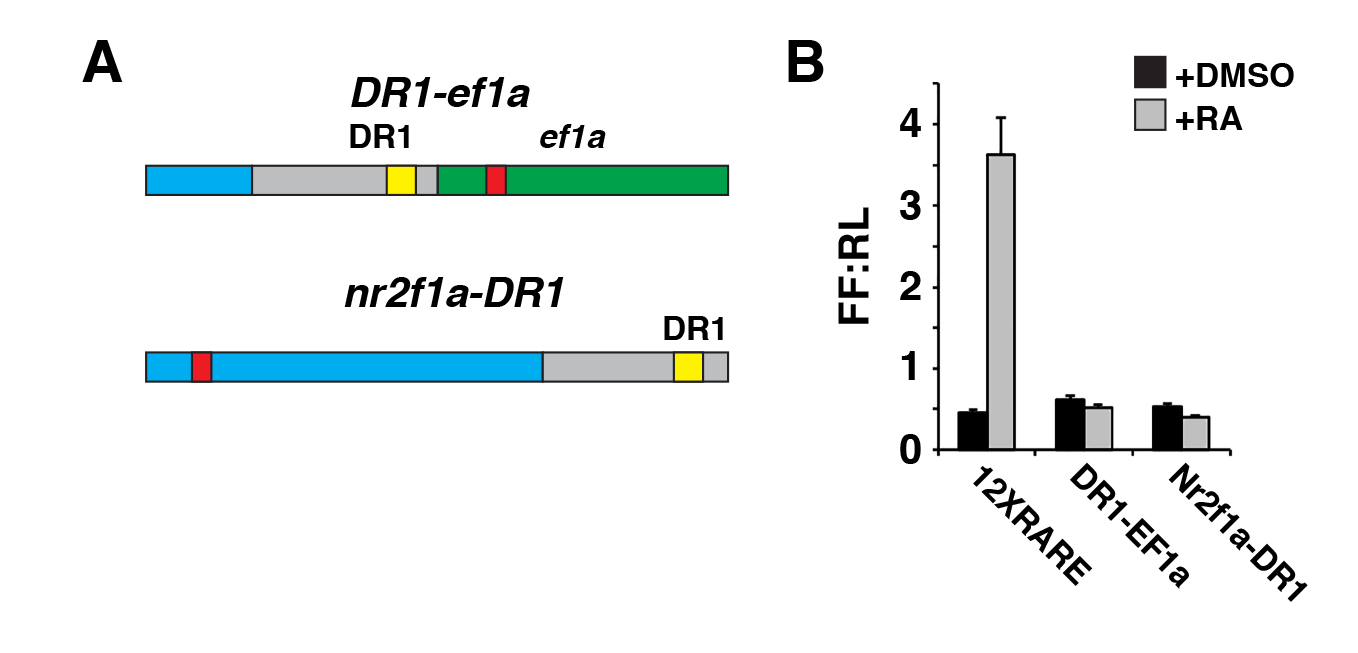

Supplement: S1 Fig — (A) Schematic of the two constructs placed into pGL3. The pGL3-DR1-ef1a construct has nucleotides -60 of the nr2f1a promoter (blue) through +105 of 5’UTR (gray), which includes the including the DR1 site (yellow box) cloned adjacent to a minimal elongation factor 1a (ef1a) promoter (green) (358 bp). The pGL3-nr2f1a-DR1 construct contains nucleotides -266 through +105 (371 bp) of the promoter and 5’UTR containing the conserved DR1 site. Blue indicates nr2f1a promoter sequences. Red boxes indicate predicted TATA boxes. (B) Luciferase assays testing RA responsiveness in HEK 293 cells. FF–firefly luciferase. RL—renilla luciferase. The previously reported pGL3-12XRARE-tk plasmid [78] was used as a positive control. (TIF) [file pgen.1007962.s001.tif]

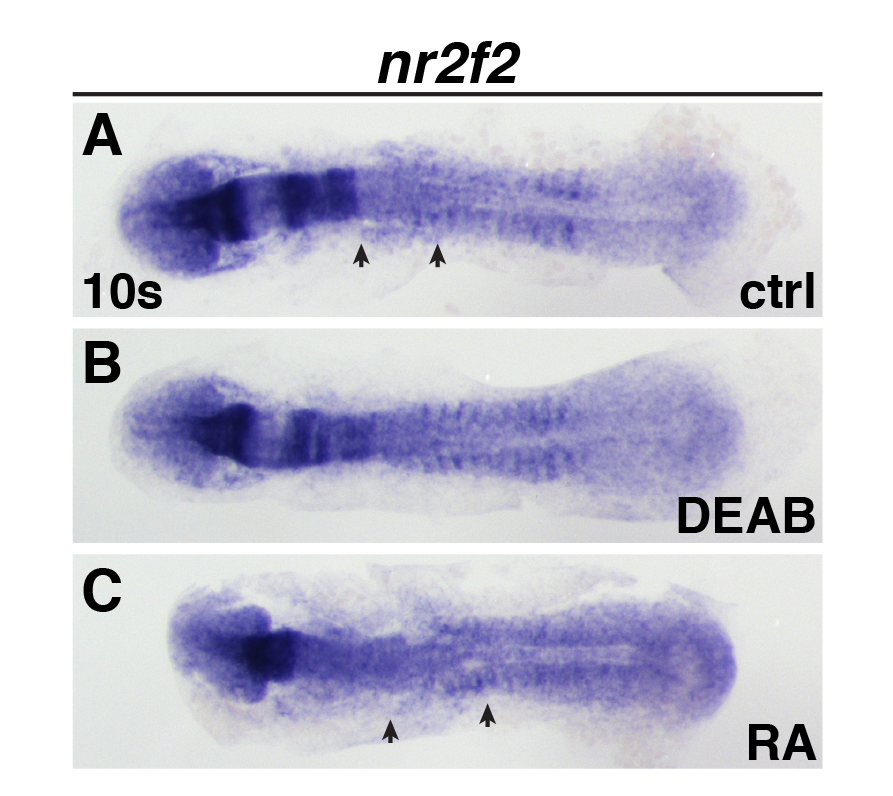

Supplement: S2 Fig — (A-C) Nr2f2 expression in the ALPM of control, DEAB-treated, and RA-treated embryos. View is dorsal with anterior left. Arrows indicated anterior and posterior limits of expression in control and RA-treated embryos. (TIF) [file pgen.1007962.s002.tif]

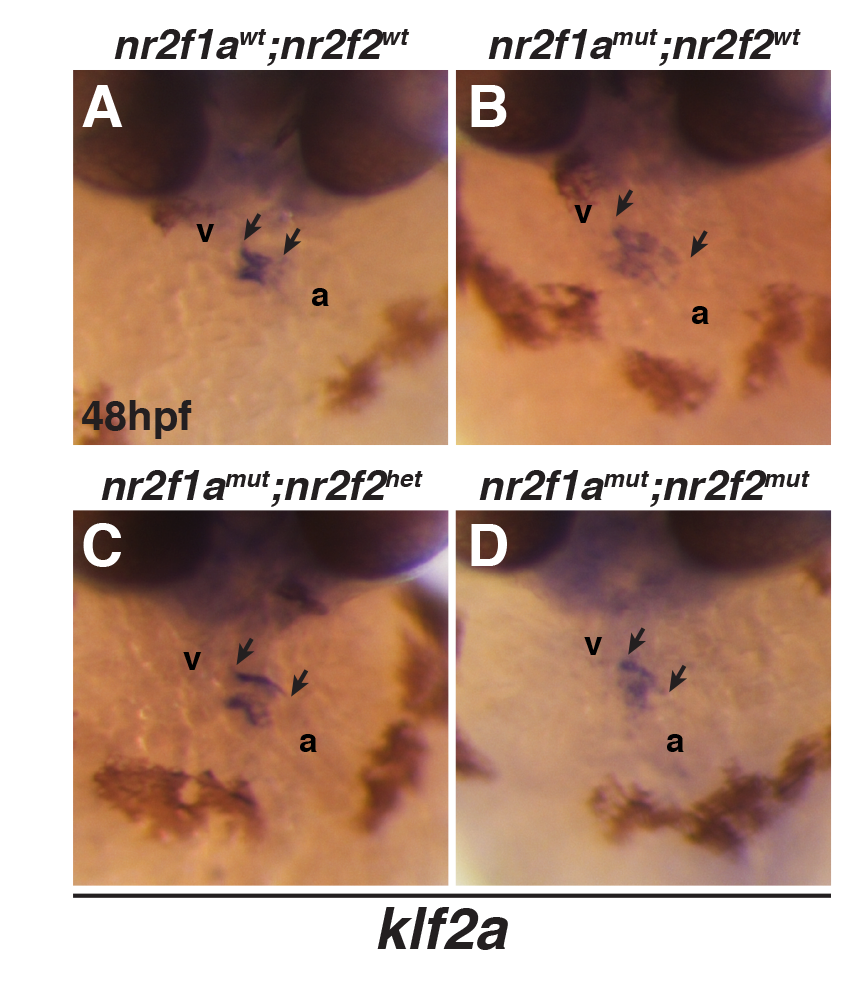

Supplement: S3 Fig — (A-D) ISH for the endocardial atrioventricular canal marker klf2a. Frontal views of hearts in nr2f1awt; nr2f2wt, nr2f1amut; nr2f2wt, nr2f1amut; nr2f2het, and nr2f1amut; nr2f2mut embryos. v–ventricle. a–atrium. Arrows indicate the length of klf2a expression within the hearts. (TIF) [file pgen.1007962.s003.tif]

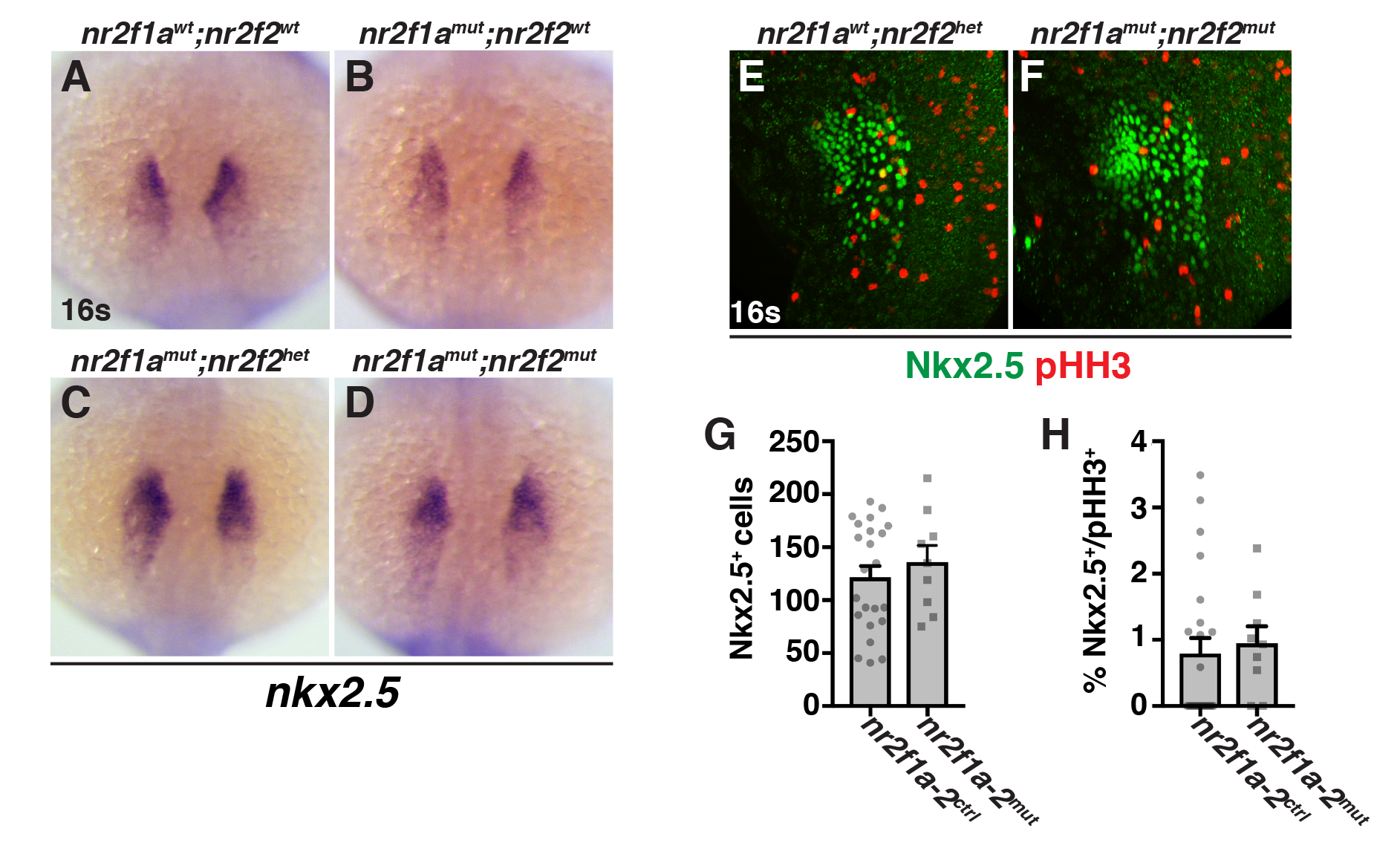

Supplement: S4 Fig — (A-D) ISH for the cardiac progenitor marker nkx2.5 in nr2f1awt; nr2f2wt, nr2f1amut; nr2f2wt, nr2f1amut; nr2f2het, and nr2f1amut; nr2f2mut embryos at the 16s stage. Dorsal view with anterior up. 160 embryos were examined with ≥9 embryos examined for each condition. Although we observed a trend in the expansion of nkx2.5 expression when assaying area of expression similar to vmhc, due to inherent variability in nkx2.5 expression and the low numbers of embryos, it was not statistically significant. (E,F) IHC for Nkx2.5 and pHH3 in nr2f1awt; nr2f2het and nr2f1amut; nr2f2mut embryos at the 16s stage. Confocal images of the ventro-lateral side of the embryo. Dorsal is right and anterior up. A single side of each embryo was used for analysis. (G) Number of Nkx2.5+ cells in control and nr2f1a; nr2f2 mutant embryos. (H) Percentage of pHH3+/Nkx2.5+ in control and nr2f1a; nr2f2 mutant embryos. For quantification of Nkx2.5+ and pHH3+/Nkx2.5+ cells, nr2f1a homozygous mutants (nr2f1amut) coupled with nr2f2 heterozygosity (nr2f2het) or nr2f2 mutant homozygosity (nr2f2mut) were analyzed together (referred to as nr2f1a-2mut), because our data suggest loss of a single WT nr2f2 allele in nr2f1a mutants produces a similar increase in ventricular CMs as double mutants. Nr2f1a-2ctrl includes any combination of nr2f1a and nr2f2 WT and heterozygous alleles. nr2f1a-2ctrl (n = 23) and nr2f1a-2mut (n = 9) for G and H. (TIF) [file pgen.1007962.s004.tif]

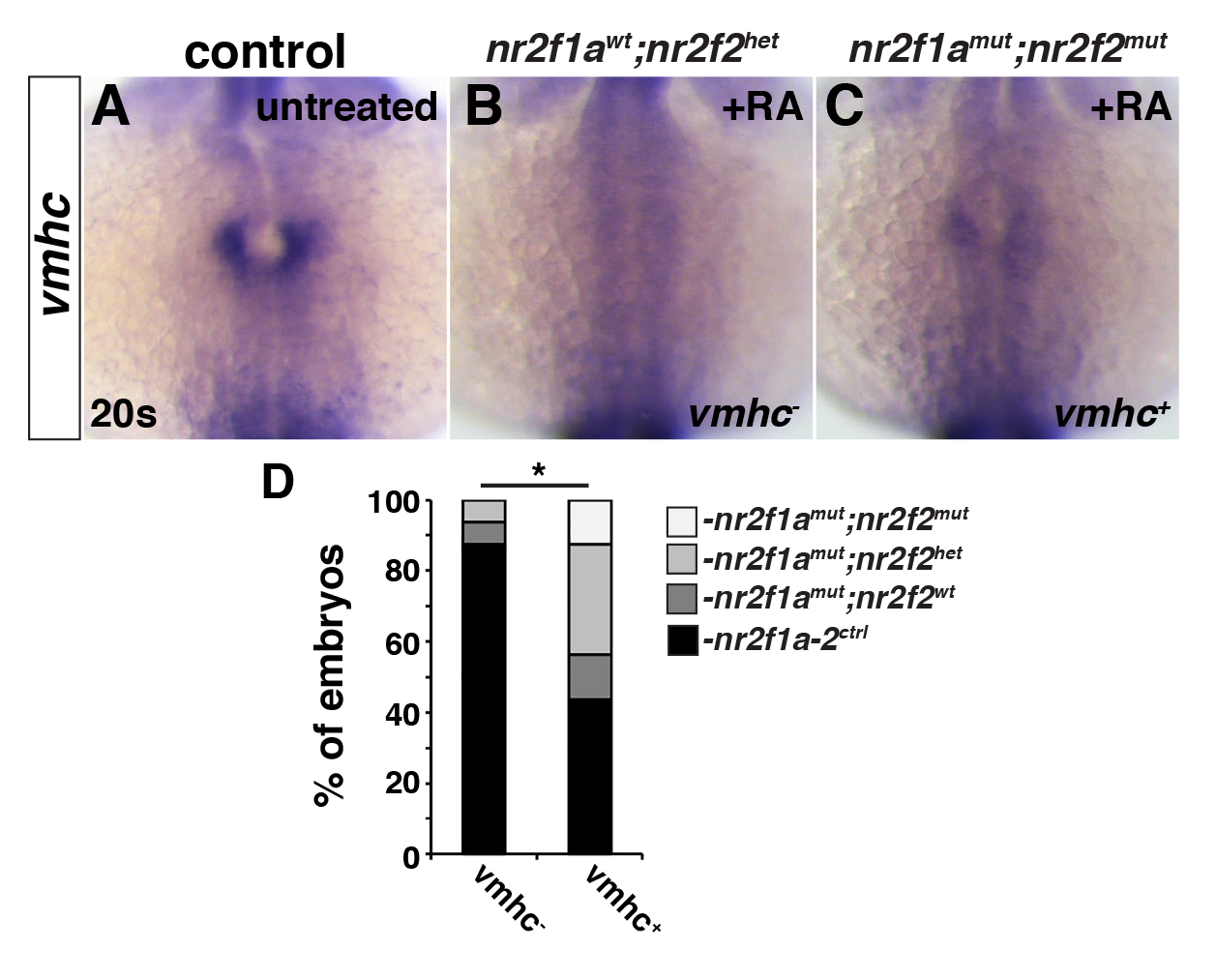

Supplement: S5 Fig — (A-C) ISH for vmhc in control (untreated), RA-treated nr2f1awt; nr2f2het, and RA-treated nr2f1amut; nr2f2mut embryos at the 20s stage. Control embryos were not genotyped. (D) Percentage of embryos with the genotypes found that lacked vmhc expression (n = 16) or had vmhc expression (n = 16). Although a RA-treated nr2f1awt; nr2f2het is shown in B, nr2f1a-2ctrl includes any combination of nr2f1a and nr2f2 WT and heterozygous alleles. Fisher’s exact test was used to compare the frequency of embryos with two nr2f1amut alleles found in each condition. (TIF) [file pgen.1007962.s005.tif]

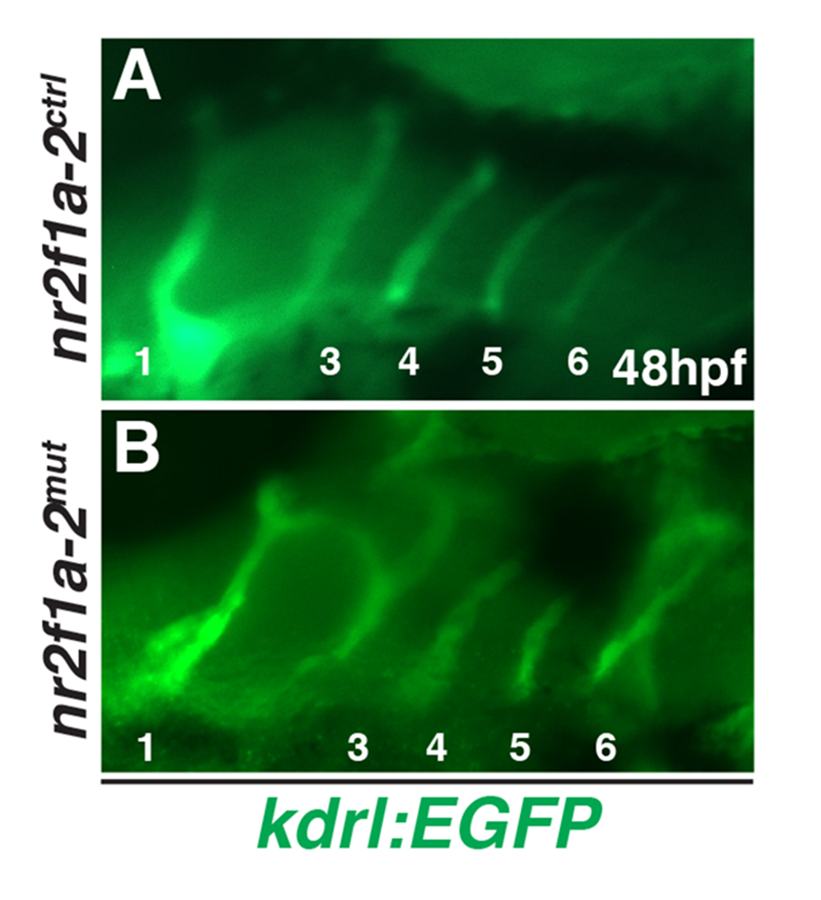

Supplement: S6 Fig — (A,B) PAAs in nr2f1a-2ctrl and nr2f1a-2mut embryos. Numbers indicated arches. Anterior is to the right. (TIF) [file pgen.1007962.s006.tif]

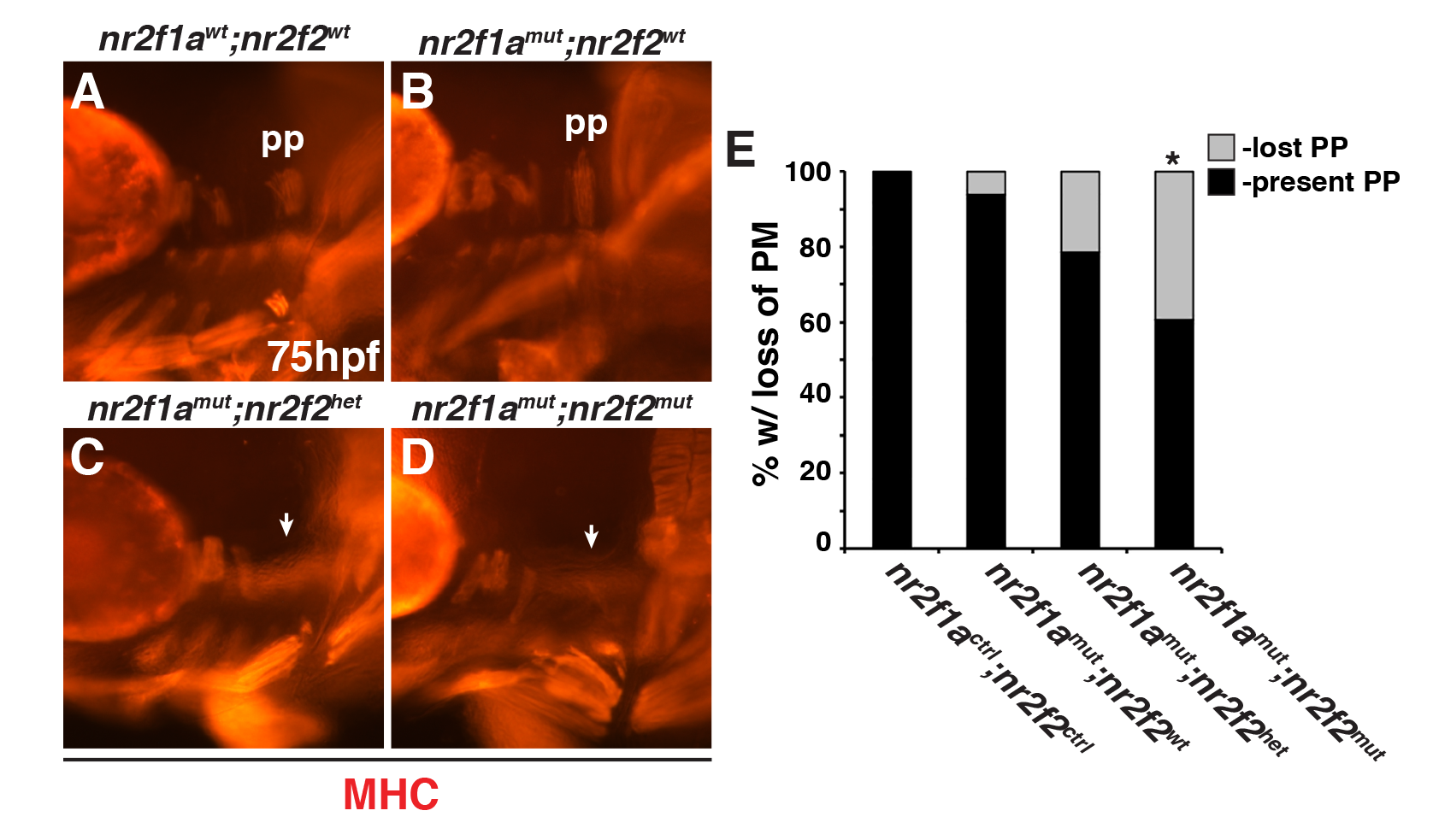

Supplement: S7 Fig — (A-D) PMs in nr2f1awt; nr2f2wt, nr2f1amut; nr2f2wt, nr2f1amut; nr2f2het, and nr2f1amut; nr2f2mut embryos at 75 hpf. Views are lateral with anterior to the left and dorsal up. (E) Percentage of nr2f1actrl; nr2f2ctrl (n = 7), nr2f1amut; nr2f2wt (n = 16), nr2f1amut; nr2f2het (n = 28), and nr2f1amut; nr2f2mut (n = 28) embryos with loss of posterior and malformed PMs at 75 hpf. (TIF) [file pgen.1007962.s007.tif]

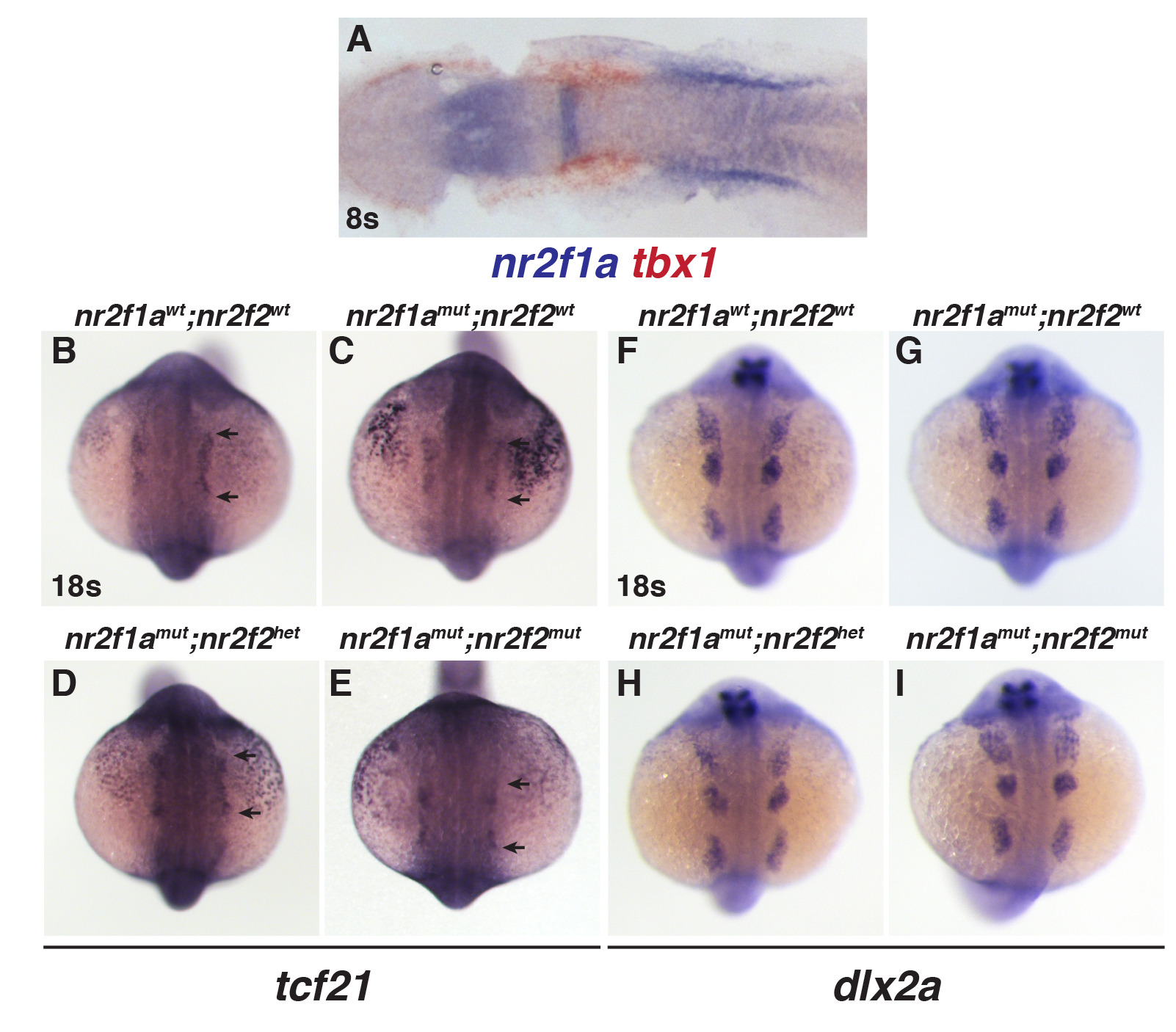

Supplement: S8 Fig — (A) ISH for tbx1 (red) and nr2f1a (blue) in the ALPM of an embryo at the 8s stage. Image is a dorsal view with anterior rightward of a flat-mounted embryo. (B-E) ISH for tcf21 in the ALPM of nr2f1awt; nr2f2wt, nr2f1amut; nr2f2wt, nr2f1amut; nr2f2het, and nr2f1amut; nr2f2mut embryos at the 18s stage. (F-I) ISH for the neural crest marker dlx2a in nr2f1awt; nr2f2wt, nr2f1amut; nr2f2wt, nr2f1amut; nr2f2het, and nr2f1amut; nr2f2mut embryos at the 18s stage. For B-I, views are dorsal with anterior up. (TIF) [file pgen.1007962.s008.tif]

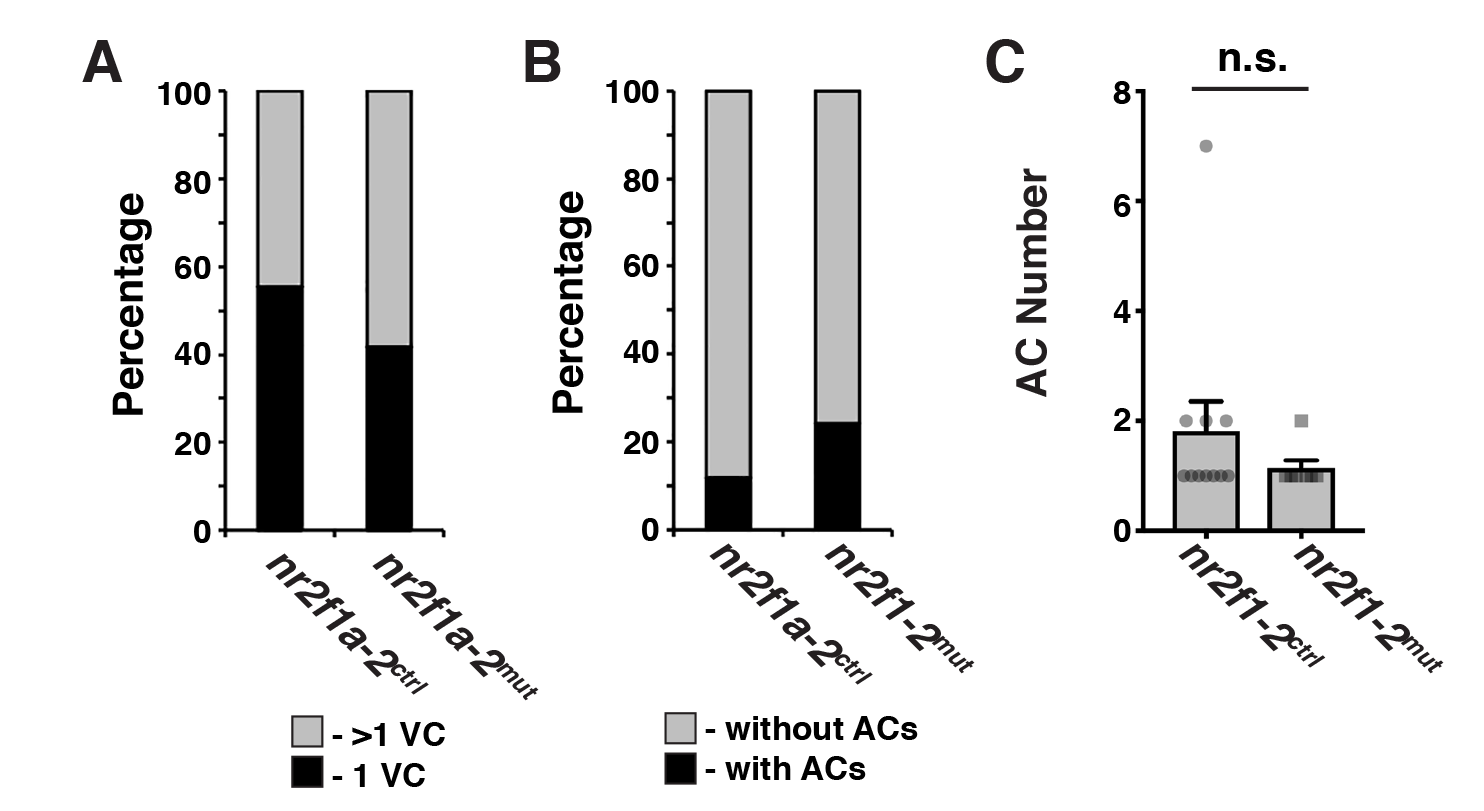

Supplement: S9 Fig — (A) Percentage of embryos with 1 and >1 ventricular CM. (B) Percentage of embryos with labeled CMs that had labeled atrial CMs. (C) Mean number of labeled atrial CMs in nr2f1a-2ctrl and nr2f1a-2mut embryos. (TIF) [file pgen.1007962.s009.tif]

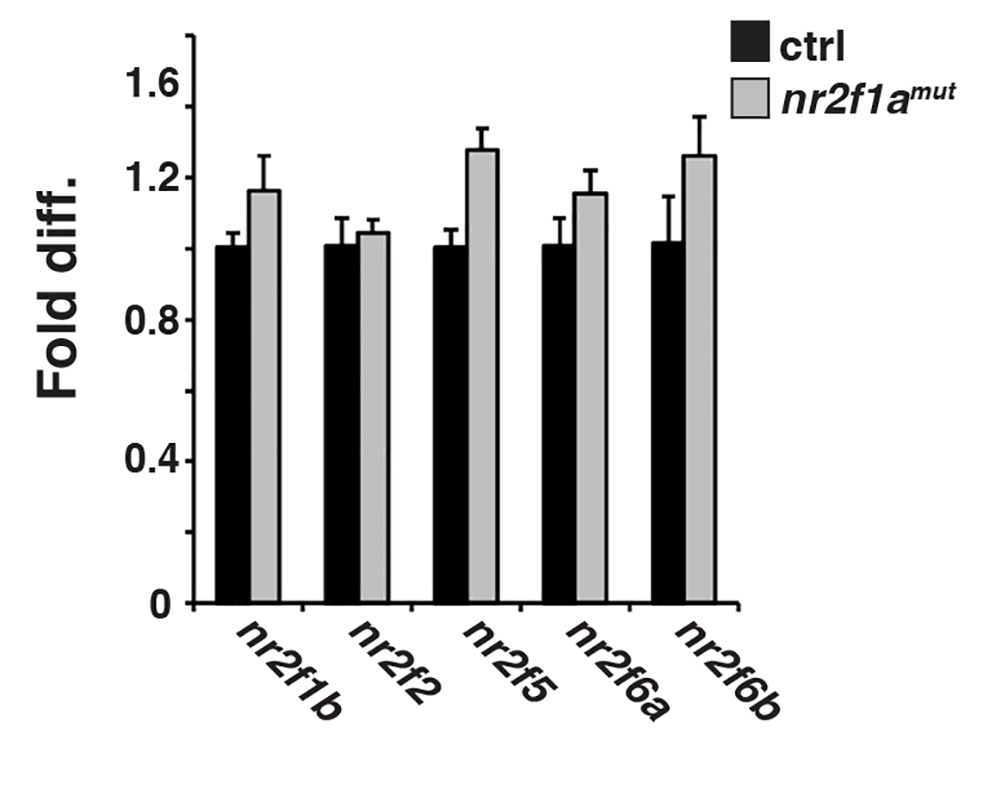

Supplement: S10 Fig — RT-qPCR for nr2f1b, nr2f2, nr2f5, nr2f6a, and nr2f6b in nr2f1a mutants at 48 hpf does not show compensatory expression. (TIF) [file pgen.1007962.s010.tif]
