# Supplementary material for: Nr2f-dependent allocation of ventricular cardiomyocyte and pharyngeal muscle progenitors
Source: PLoS Genet. 2019 Feb 5;15(2):e1007962. doi: 10.1371/journal.pgen.1007962 (PMC6377147; doi:10.1371/journal.pgen.1007962)
Supplement: S1 Table — (DOCX) [file pgen.1007962.s011.docx]

**S1 Table. Primers sequences.**

| **Application** | **Primer Name** | **Sequence** |
| --- | --- | --- |
| **RT-qPCR** | *β-actin* forward | TACAGCTTCACCACCACAGC |
|  | *β-actin* reverse | AGGAAGGAAGGCTGGAAGAG |
|  | *nr2f1a* forward | TCAAGCGGCAAGCACTATGG |
|  | *nr2f1a* reverse | TTCGGTTGGGTTGGAGGCATTC |
|  | *nr2f1b* forward | CAGACATCCCGCACATTGAAG |
|  | *nr2f1b* reverse | CCAGCCGAACGAAGAAAAGC |
|  | *nr2f2* forward | CGCCTTTATGGACCACATTAGG |
|  | *nr2f2* reverse | GCTCTATGACCGAAGACGAGACTG |
|  | *nr2f5* forward | TTGACCAGCACCATCGGAAC |
|  | *nr2f5* reverse | ATACATTGGGCTCCATAGCGGGAC |
|  | *nr2f6a* forward | CGGTGAGGATGATAAGGGATGTG |
|  | *nr2f6a* reverse | CCACGCTGAACTGCTTCTTTACG |
|  | *nr2f6b* forward | TGGTGGACTGTGTTGTGTGTGG |
|  | *nr2f6b* reverse | TTCTTTCCTCATACCGACCCG |
| **CHIP-qPCR** | *cyp26a1* DR5 forward | GAGAGTTTGGAGCCGCTTCT |
|  | *cyp26a1* DR5 reverse | CGCAGGTCTCCTCCTGTTA |
|  | *nr2f1a* DR1 forward | GATGCAGGGTTCTGACCAGT |
|  | *nr2f1a* DR1 reverse | GCGTTTTAACGGGAGTACCA |
|  | *nr2f1a*-ctrl forward | CACCGCTTGATTCCAAAAA |
|  | *nr2f1a*-ctrl reverse | ACCCTGTCTGCCAATGTGTT |
