# Supplementary material for: Nr2f-dependent allocation of ventricular cardiomyocyte and pharyngeal muscle progenitors
Source: PLoS Genet. 2019 Feb 5;15(2):e1007962. doi: 10.1371/journal.pgen.1007962 (PMC6377147; doi:10.1371/journal.pgen.1007962)
Supplement: S2 Table — (DOCX) [file pgen.1007962.s012.docx]

**S2 Table. Antibodies used.**

|  | **Antibody** | **Supplier** | **Product** | **Procedure** | **Dilution** |
| --- | --- | --- | --- | --- | --- |
| **Primary** | rabbit anti-RCFP | Clontech | 632475 | IHC | 1:1000 |
|  | rabbit anti-DsRed2/mCherry | Clontech | 632496 | IHC | 1:1000 |
|  | mouse anti-MF20 | DSHB | MF20 (supernatant) | IHC | 1:10 |
|  | mouse anti-S46 | DSHB | S46 (supernatant) | IHC | 1:10 |
|  | rabbit anti-GFP | Abcam | Ab290 | ChIP | N/A |
|  | rabbit anti-GFP | Abcam | Ab290 | IHC | 1:500 |
|  | rabbit anti-Nkx2.5 | GeneTex | 128357 | IHC | 1:250 |
|  | mouse anti-phosphoHistone H3 | Abcam | Ab14955 | IHC | 1:1000 |
| **Secondary** | goat anti-mouse IgG1 FITC | Southern Biotech | 1070–02 | IHC | 1:100 |
|  | goat anti-rabbit IgG-TRITC | Southern Biotech | 4050–03 | IHC | 1:100 |
|  | goat anti-rabbit IgG-FITC | Southern Biotech | 4050-02 | IHC | 1:100 |
|  | goat anti-mouse IgG2b TRITC | Southern Biotech | 1090–03 | IHC | 1:100 |
|  | goat anti-rabbit IgG(H+L), Alexa Fluor^®^ 488 | Southern Biotech | 4050-30 | IHC | 1:100 |
|  | goat anti-mouse IgG1 TRITC | Southern Biotech | 1070–03 | IHC | 1:100 |
